# Identification and Validation of a Prognostic Prediction Model of m6A Regulator-Related LncRNAs in Hepatocellular Carcinoma

**DOI:** 10.3389/fmolb.2021.784553

**Published:** 2021-12-20

**Authors:** Chen Jin, Rui Li, Tuo Deng, Jialiang Li, Yan Yang, Haoqi Li, Kaiyu Chen, Huihua Xiong, Gang Chen, Yi Wang

**Affiliations:** ^1^ Department of Epidemiology and Biostatistics, School of Public Health and Management, Wenzhou Medical University, Wenzhou, China; ^2^ Department of Oncology, Tongji Hospital, Huazhong University of Science and Technology, Wuhan, China; ^3^ Department of Hepatobiliary Surgery, The First Affiliated Hospital of Wenzhou Medical University, Wenzhou, China; ^4^ Key Laboratory of Diagnosis and Treatment of Severe Hepato-Pancreatic Diseases of Zhejiang Province, The First Affiliated Hospital of Wenzhou Medical University, Wenzhou, China

**Keywords:** hepatocellular carcinoma, m6A regulators, long non-coding RNA, prognosis prediction, therapy

## Abstract

Hepatocellular carcinoma (HCC) is a highly invasive malignancy prone to recurrence, and patients with HCC have a low 5-year survival rate. Long non-coding RNAs (lncRNAs) play a vital role in the occurrence and development of HCC. N6-methyladenosine methylation (m6A) is the most common modification influencing cancer development. Here, we used the transcriptome of m6A regulators and lncRNAs, along with the complete corresponding clinical HCC patient information obtained from The Cancer Genome Atlas (TCGA), to explore the role of m6A regulator-related lncRNA (m6ARlnc) as a prognostic biomarker in patients with HCC. The prognostic m6ARlnc was selected using Pearson correlation and univariate Cox regression analyses. Moreover, three clusters were obtained *via* consensus clustering analysis and further investigated for differences in immune infiltration, immune microenvironment, and prognosis. Subsequently, nine m6ARlncs were identified with Lasso-Cox regression analysis to construct the prognostic signature m6A-9LPS for patients with HCC in the training cohort (*n* = 226). Based on m6A-9LPS, the risk score for each case was calculated. Patients were then divided into high- and low-risk subgroups based on the cutoff value set by the X-tile software. m6A-9LPS showed a strong prognosis prediction ability in the validation cohort (*n* = 116), the whole cohort (*n* = 342), and even clinicopathological stratified survival analysis. Combining the risk score and clinical characteristics, we established a nomogram for predicting the overall survival (OS) of patients. To further understand the mechanism underlying the m6A-9LPS-based classification of prognosis differences, KEGG and GO enrichment analyses, competitive endogenous RNA (ceRNA) network, chemotherapeutic agent sensibility, and immune checkpoint expression level were assessed. Taken together, m6A-9LPS could be used as a precise prediction model for the prognosis of patients with HCC, which will help in individualized treatment of HCC.

## Introduction

According to the latest data released by the International Agency for Research on Cancer (IARC), hepatocellular carcinoma (HCC) is one of the most common malignancies worldwide, with a high mortality rate and an increasing annual morbidity trend (Siegel et al., 2020), posing a tremendous threat to public health (Lin et al., 2020). In recent years, significant advances have been made in terms of treatments such as partial hepatectomy, liver transplantation, interventional surgery, and systemic treatment for HCC (Wu et al., 2020), which significantly improves the prognosis of patients with early-stage HCC. However, the long-term outcome of late-stage HCC, which is found in half of the patients at the time of diagnosis, is still devastating (Jung et al., 2017). At present, extensively applied staging systems using clinicopathological factors, such as histological grade, lymph node metastasis, and tumor-node-metastasis (TNM), provide relatively vague predictions in evaluating the options for treatment and the prognostic outcomes for patients with HCC (Cao et al., 2014; Yoo et al., 2018). In fact, there is a lack of a highly sensitive and specific tumor signature to accurately evaluate the prognosis of patients. Therefore, it is imperative to identify novel and precise biomarkers, for both prognosis and improvement of individualized treatments for patients.

RNA modification is the post-transcriptional regulation of gene expression, which is widely present in various RNA types (Agris et al., 2017; Roundtree et al., 2017). To date, more than 100 types of RNA modifications have been identified; among them, RNA methylation accounts for more than 60% of all modifications (Zhang S. et al., 2020). N6-methyladenosine (m6A), the most common type of RNA methylation modification in eukaryotes, plays an important role in the biological processes of immunity, metabolism, proliferation, and apoptosis (Meyer and Jaffrey 2017; Huang et al., 2018). The methylation level of m6A in tumors is mainly modulated by three types of regulators: methyltransferases, demethylases, and methyl-binding proteins (Robinson et al., 2019; Wang et al., 2021). Various studies have shown that the abnormal regulation of m6A is correlated with the occurrence and development of various human tumors such as cervical cancer, breast cancer, prostate cancer, lung cancer, pancreatic cancer, hepatocellular carcinoma, and glioma (Zhang B. et al., 2020; Ma X. et al., 2020; Dong and Cui 2020; Hou et al., 2020; Yuan et al., 2020; Zhao et al., 2020). For example, the impaired autophagic degradation of ARHGAP5-AS1 by METT12 promotes chemoresistance in gastric cancer patients, which is associated with a poor prognosis (Zhu et al., 2019); LINC00662 regulates the radioresistance of oral squamous carcinoma cells by affecting AK4 (Chen Y. et al., 2020); upregulation of PVT1 mediated by ALKBH5 affects the prognosis of osteosarcoma (Chen S. et al., 2020).

Long non-coding RNA (lncRNA) is a non-protein-encoding RNA with a transcript length more than 200 bp. LncRNAs can perform *cis* or *trans* transcriptional regulation or RNA molecular regulation. It is known that lncRNAs regulate various aspects of tumors (Kung and Lee 2013; Tran et al., 2016; Kopp and Mendell 2018). For example, CHRF is an lncRNA associated with the epithelial-to-interstitial transformation (EMT), which participates in the occurrence and development of gastric cancer (Gong J. et al., 2020); upregulation of the TUC338 may activate the MAPK pathway to promote lung cancer invasion (Zhang et al., 2018); many studies have reported that lncRNAs are related to the progression and prognosis of liver cancer (Li et al., 2018; Pan et al., 2019; Hong et al., 2020). However, few studies have explored the relationship between lncRNA and the expression of m6A regulators with the occurrence or development of HCC. In addition to the potential applications, value, and mechanisms of lncRNAs regulating the expression of m6A regulators, the relationship between lncRNAs and the prognosis of patients with HCC is still unclear.

Therefore, in this study, we collected transcriptomic data and clinicopathological data of patients with HCC and conducted a series of bioinformatic analyses to understand the m6A regulator-related lncRNA expression and its impact in HCC, and to shed light on the underlying mechanisms for the prognosis of patients. The results of this study may help identify biomarkers that could be used as novel therapeutic targets for HCC.

## Materials and Methods

### Data Collection and Pre-processing to Identify Prognostic lncRNAs Associated With m6A Regulators

FPKM-masked sequencing data of 374 primary hepatocyte malignant tissues and 50 normal tissues were obtained from The Cancer Genome Atlas (TCGA; https://portal.gdc.cancer.gov) database and annotated with human gene annotation files from the Ensembl official website (http://asia.ensembl.org/index.html; GRCh38.103. chr.gtf.gz). GTF files (GRCh38.103. chr.gtf.gz) were used to annotate and distinguish the mRNAs and lncRNAs for subsequent analysis. Corresponding clinicopathological data were downloaded concomitantly. Patients with pathologically confirmed HCC and complete clinical information were included in this study; those with a survival time inferior or equal to 30 days were excluded to avoid any bias originating from demise due to unrelated factors. A total of 342 HCC cases were included in the subsequent analysis.

By systematically searching and reviewing representative m6A-related literature, 23 universally acknowledged m6A regulators, namely, 8 methyltransferases, 2 demethylases, and 13 binding proteins, were selected. The details of these genes ^[14, 31–35]^ are listed in Supplementary Table S1. We calculated the correlation coefficient between the expression levels of m6A regulators and the expression level of lncRNA and selected the one with |Pearson *r*| > 0.4 and *p* < 0.001 as the m6A regulator-related lncRNA (m6ARlnc). Then, the expression matrix of the m6ARlnc genes was extracted. A flow chart of this study is illustrated in Supplementary Figure S1.

To identify prognostic m6ARlncs, univariate Cox regression analysis was conducted to screen the differentially expressed lncRNAs between the tumor and normal groups, with significance set at *p* ≤ 5 × 10^−5^, using the *limma* R package.

### Comprehensive Analysis of Patients With HCC Based on the Consensus Clustering of Prognostic m6ARlnc

Based on the differentially expressed prognostic m6ARlncs in tumor and normal tissues, unsupervised consensus clustering was performed using the *ConsensusClusterPlus* R package. Based on the similarity between the expression levels of prognostic m6ARlncs and the proportion of fuzzy clustering measurements, the cluster number with the best stability was determined. In addition, CIBERSORT analysis was performed to determine the proportion of 22 types of immune cells in each sample. The ESTIMATE algorithm was used to evaluate the tumor microenvironment (TME) score (including stromal cell, immune cell, and comprehensive scores).

### Establishment and Validation of an m6ARlncs Prognostic Prediction Model

To fully understand the predictive value of prognostic m6ARlnc, 342 HCC cases were randomly divided into a training cohort (*n* = 226) and a validation cohort (*n* = 116) in a 2:1 ratio. In this study, all subsequent analyses were performed in the training cohort, except for model validation, hierarchical analysis of the model, and enrichment analysis of the signature, which were performed on the entire cohort. First, the *glmnet*, *survminer*, and *survival* R packages were used to conduct Lasso-Cox regression analysis to construct a prognosis prediction model of patients with HCC by screening the prognostic m6ARlncs. The model was optimized by cross-validation, and lncRNAs used to construct the prognostic signature were screened. A survival plot was constructed using the *survival* R package. The risk score of each patient was calculated using the following formula:

$$Risk\;score=\sum_{i=1}^{n}Coef_{i}\times X_{i}$$

*Coef*_*i*_ represents the coefficient of the corresponding m6ARlnc from the Cox regression model, and *X*_*i*_ is the expression level of m6ARlnc. The X-tile software was used to determine the optimal cutoff value of the risk score for the training cohort, and the same cutoff value was used in the validation and entire cohorts. Thus, all patients were divided into high- and low-risk groups. Second, Kaplan–Meier (K-M) survival curve analysis was conducted to compare the survival rates between groups using the *survival* R package.

The receiver operator characteristic (ROC) curves for 1, 3, and 5 years were constructed using the *timeROC* R package to evaluate the predictive accuracy of this signature, and the areas under the curve (AUC) were calculated. In addition, to confirm that the signature has consistent predictions across patients with different clinical characteristics such as sex, age, and tumor status, we conducted subgroup stratification survival analysis based on clinicopathological features using the K-M plot.

### Construction of a Nomogram Integrating the Prognostic Signature and Clinical Characteristics

To improve the clinical application of the prognostic signature, we constructed a comprehensive prediction model combining the signature risk score and clinicopathological factors. First, we conducted a univariate Cox regression analysis, and variables with *p* < 0.0001 were subsequently included in the multivariate Cox regression analysis to screen independent factors in predicting the prognosis of patients with HCC. Second, we evaluated whether there were differences among clinicopathological factors, classification characteristics of prognostic m6ARlnc, stromal cell score, immune cell score, comprehensive score, and risk score between the differential expression of lncRNAs in the prognostic signature. The stromal cell, immune cell, and comprehensive scores were calculated using the ESTIMATE algorithm. Finally, a nomogram was established, according to the independent prognostic factors, to predict overall survival (OS) of patients using the *rms* R package.

### Identification of Enriched Signaling Pathways and Function Between High- and Low-Risk Patients Classified by Prognostic Signature

To identify potential signaling pathways related to high- and low-risk patients determined by prognostic signature, we performed a gene set enrichment analysis (GSEA) using the GSEA software (version: 4.1.0). The gene set *c2. cp.kegg.v7.4. symbols.gmt* was downloaded from Kyoto Encyclopedia of Genes and Genomes (KEGG) enrichment analysis, and the gene set used for Gene Ontology (GO) enrichment analysis was *c5. go.bp.v7.3. symbols.gmt*.

### Construction of a ceRNA Network and Functional Enrichment Analysis

To explore the regulatory relationship between lncRNA and m6A regulators, constructing a ceRNA network is a good choice. The miRcode database (http://www.mircode.org/) was used to predict miRNAs that interact with prognostic m6ARlncs. To identify the target mRNAs, the relationship between miRNA and target mRNA was characterized using miRDB (http://www.mirdb.org/), miRTarBase (https://mirtarbase.cuhk.edu.cn/), and TargetScan (http://www.targetscan.org/). In addition, the Cytoscape (version 3.6.0) software was used to visualize the lncRNA–miRNA–mRNA ceRNA network. Subsequently, GO and KEGG enrichment analyses of target mRNAs were performed using the *clusterProfiler* R package to explore the potential signaling pathways of m6ARlncs.

### Evaluation of Chemotherapeutic Agent Sensitivity and Expression of Immune Checkpoints Among Patients With HCC in High- and Low-Risk Groups

To predict the sensitivity of high- and low-risk patients to chemotherapeutic agents, we used the *pRRophetic* (https://github.com/paulgeeleher/pRRophetic) R package to predict the half-maximal inhibitory concentration (IC50) of chemotherapeutic agents for each patient. The R package was based on pre-treatment gene expression and drug sensitivity data of cancer cell lines to predict the chemotherapeutic response (Geeleher et al., 2014). Thirty-three immune checkpoints (Supplementary Table S2) were used to evaluate the performance of the model for immunotherapy and to analyze the differences in immune checkpoint expression levels between high- and low-risk groups.

### Statistical Analysis

The Wilcoxon rank-sum test was used to compare the differences between two groups of continuous variables, and the Kruskal–Wallis test was used to examine the difference in TEM scores between the different subtypes. The chi-square test was used to examine the expression differences between lncRNAs. The K-M curve, combined with the log-rank test, was used to evaluate the OS of the different groups. Independent predictive factors were identified using Cox proportional hazards regression analysis. Based on the results of multivariate Cox analysis, a nomogram was established and evaluated for consistency using the C-index and calibration curve. All statistical analyses were carried out using the R software (version 3.6.3). Unless indicated otherwise, *p* < 0.05 was considered statistically significant.

## Results

### Identification of m6ARlnc in Patients With HCC

**FIGURE 1:**
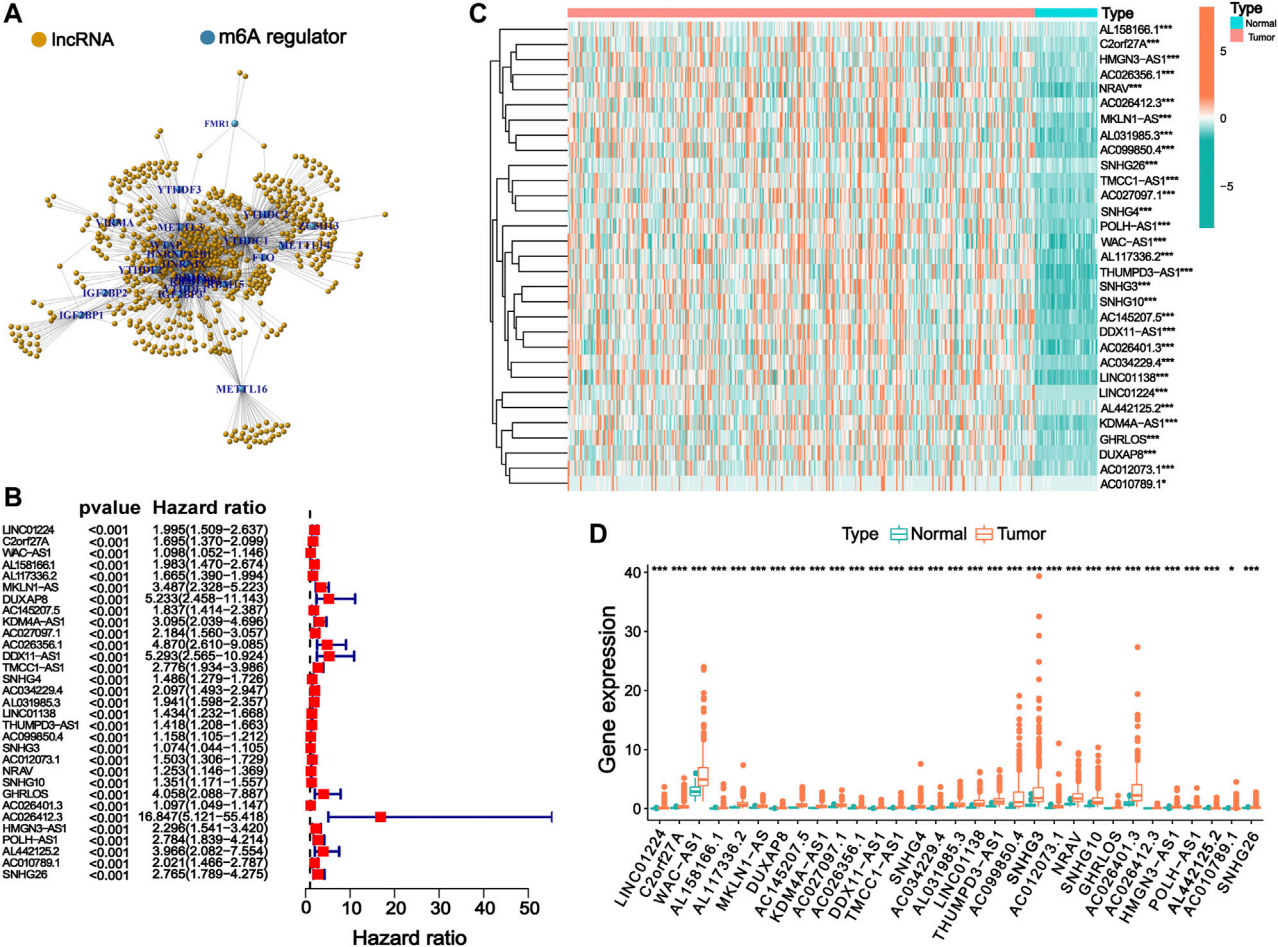
Identification of m6ARlnc and prognostic m6ARlnc. **(A)** The co-expression network between m6A regulators and m6A regulator-related lncRNAs. **(B)** Forest plot of predictive ability of 31 prognostic lncRNAs associated with m6A regulators. **(C)** The heatmap of the expression of 31 m6A regulator-related prognostic lncRNAs in cancer and normal groups in the TCGA cohort (green: low expression level, red: high expression level). **(D)** Box plot graph of the expression of 31 prognostic lncRNAs related to m6A regulators in the TCGA cohort (green: low expression level, red: high expression level; *p*-value was: ^***^ *p* < 0.001, ^*^ *p* < 0.05).

A total of 644 lncRNAs that were significantly correlated with 23 m6A regulators were identified in the TCGA dataset and defined as m6ARlnc. The co-expression network between these 23 m6A regulators and the related lncRNAs is shown in Figure 1A. We conducted a univariate Cox regression analysis, and the results indicated that out of these 644 lncRNAs, 31 were associated with the OS of patients with HCC (Figure 1B). We further compared the expression levels of these 31 lncRNAs between tumor and normal tissues and found that the tumor group presented a significantly higher expression level (Figures 1C,D).

### Association of Consensus Clustering of Prognostic m6ARlnc With Characteristics and Survival of Patients With HCC

**FIGURE 2:**
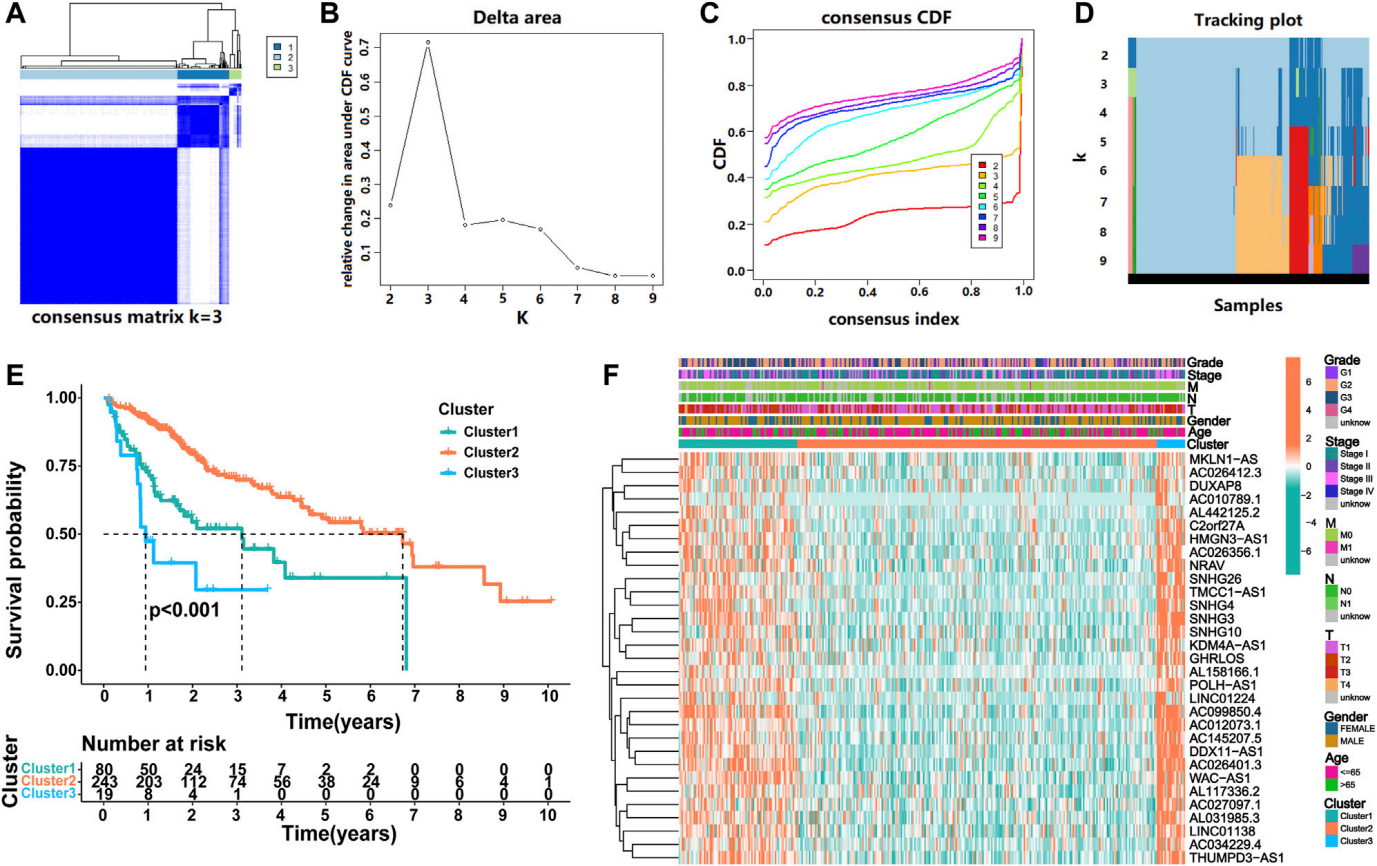
Consensus clustering of prognostic lncRNAs related to m6A regulators. **(A)** Consensus clustering matrix for *k* = 3. **(B)** Consensus clustering cumulative distribution function (CDF), **(C)** the relative change of area under the CDF curve and **(D)** tracking plot from *k* = 2 to 9. **(E)** Kaplan–Meier curve of OS for three clusters in HCC. **(F)** The heatmap for expression levels of 31 m6A regulator-related prognostic lncRNAs in the TCGA cohort, based on the relationship between three clusters and clinicopathological features.

To identify heterogeneity, unsupervised consensus clustering was performed on all samples based on the expression levels of prognostic m6ARlncs. We determined that *k* = 3 had the best clustering stability among the range *k* = 2 to 9 (Figures 2A–D). Thus, patients with HCC were divided into three subgroups: Cluster1 (*n* = 80), Cluster2 (*n* = 243), and Cluster3 (*n* = 19). To evaluate the prognostic value of m6ARlncs, survival analysis was performed on the different subgroups; significant differences were observed in the clinical outcomes. The OS of Cluster2 was the highest, while that of Cluster3 was the lowest (Figure 2E). The heatmap shows the differences in the expression of prognostic m6ARlncs in different subgroups, and the expression levels of most prognostic m6ARlncs were elevated in Cluster3; contrastingly, their expression levels were lowest in Cluster2. The differences in the expression of prognostic m6ARlncs in concomitance of various clinicopathological factors are also described in the above-mentioned heatmap. There was no difference in the expression of these lncRNAs among the clinicopathological factors (Figure 2F). Our results indicate a close relationship between the aggregation subgroup defined by the expression levels of prognostic m6ARlncs and the heterogeneity of patients with HCC.

### Immunocyte Infiltration Landscape of Prognostic m6ARlncs

**FIGURE 3:**
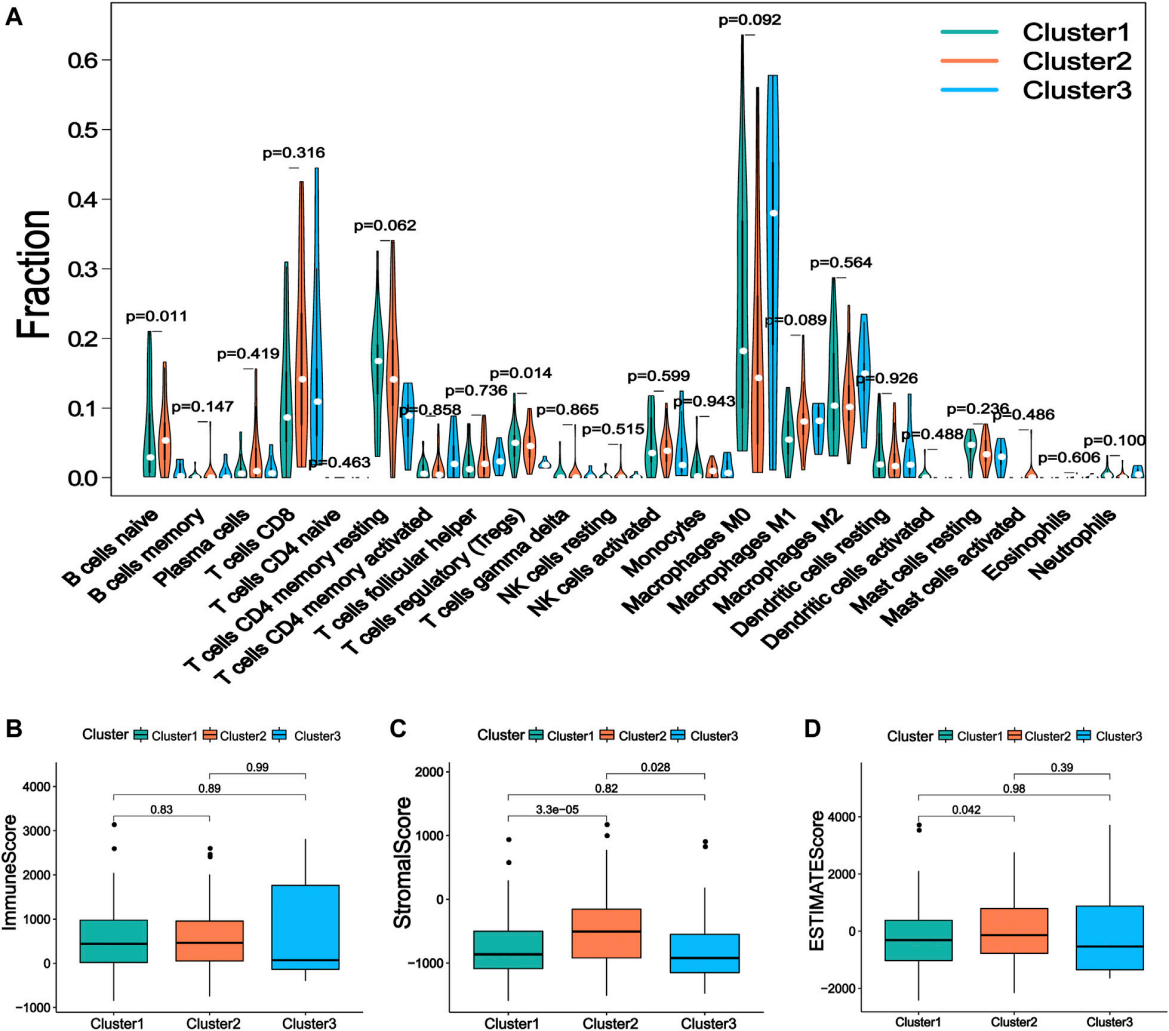
The immune characteristics of the prognostic lncRNA patterns associated with three m6A regulators. **(A)** The difference of 22 kinds of immune cell infiltration levels under the three kinds of prognosis m6A regulator-related lncRNA. **(B)** The expression of immune score, **(C)** stromal cell score, and **(D)** comprehensive score in lncRNA patterns of three kinds of prognosis m6A regulator-related lncRNA.

Increasing evidence shows that m6ARlncs are involved in the tumor immune microenvironment. CIBERSORT analysis showed that T cells regulatory (*p* = 0.014) and B cells naive (*p* = 0.011) were significantly enriched among the three subgroups (Figure 3A). Then, the stromal cell score, immune cell score, and comprehensive score of each sample were obtained using the ESTIMATE algorithm. Results revealed that the stromal cell score of Cluster2 was higher than that of Cluster1 (*p* < 0.001) and Cluster3 (*p* = 0.028), and the comprehensive score of Cluster2 was also higher than Cluster1 (*p* = 0.042), indicating that Cluster2 contained more immune-related cells and tumor cells of lower purity (Figures 3B–D).

### Establishment of a Prognostic Signature Based on m6ARlncs and Performance Evaluation and Validation of the Signature

**FIGURE 4:**
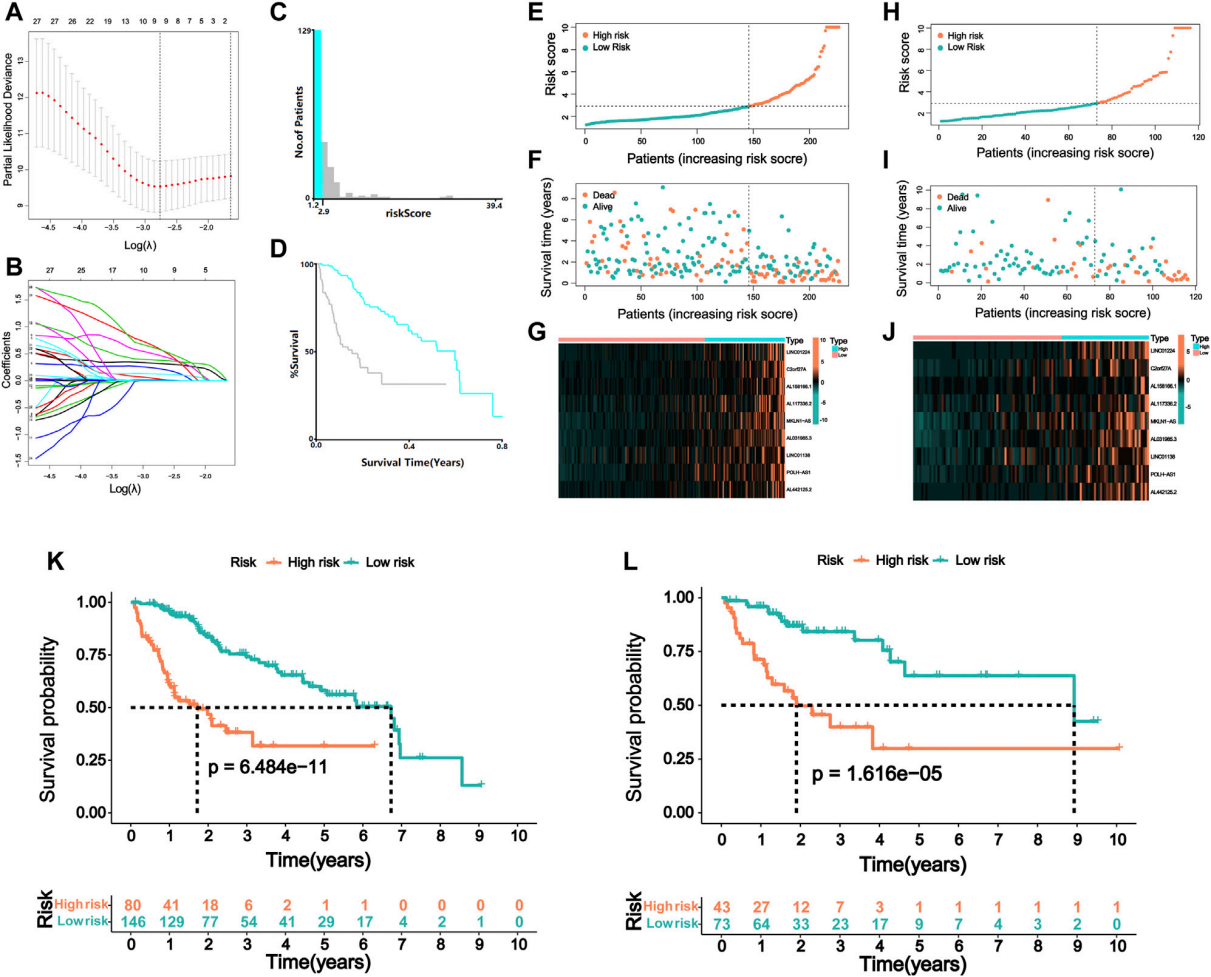
Risk model of m6A regulator-related lncRNA. **(A)** Lasso Cox regression analysis of 31 m6A regulator-related prognostic lncRNAs. **(B)** Partial likelihood deviation for different number of variables. **(C)** The optimal cutoff value of risk score identified by X-tile analysis. **(D)** Kaplan–Meier curve of HCC patients with the best cutoff value in X-tile analysis. The picture is generated by X-tile software. **(E)** The distribution of risk scores and **(F)** survival status of HCC patients in the training cohort. **(G)** The expression matrix of nine lncRNAs included in the model in the training cohort of HCC patients. **(H)** The distribution of risk scores and **(I)** survival status of HCC patients in the validation cohort. **(J)** The expression matrix of nine lncRNAs included in the model in the validation cohort of HCC patients. Kaplan–Meier curve of OS of high-risk and low-risk HCC patients in the **(K)** training and **(L)** validation cohort.

Nine lncRNAs (LINC01224, C2orf27A, AL158166.1, AL117336.2, MKLN1-AS, AL031985.3, LINC01138, POLH-AS1, and AL442125.2) were finally selected to construct a prognostic signature (m6A-9LPS) using LASSO Cox regression (Figures 4A,B). The risk score for each HCC patient was calculated based on the expression levels and coefficients of these nine lncRNAs, using the following equation (Table 1):

$$\begin{array}{l} \text{Risk}\;\text{score}=\left(0.3222\times \text{LINC}01224\;\text{expression}\;\text{level}\right)+\left(0.0363\times \text{C}2\text{orf}27\text{A}\;\text{expression}\;\text{level}\right)+\left(0.2281\times \text{AL}158166\text{.}1\;\text{expression}\;\text{level}\right)+\left(0.1265\times \text{AL}117336\text{.}2\;\text{expression}\;\text{level}\right)+\left(0.4807\times \text{MKLN}1-\text{AS}\;\text{expression}\;\text{level}\right)+\left(0.3838\times \text{AL}031985\text{.}3\;\text{expression}\;\text{level}\right)+\left(0.0123\times \text{LINC}01138\text{ expression}\;\text{level}\right)+\left(0.6103\times \text{POLH}-\text{AS}1\;\text{expression}\;\text{level}\right)+\left(0.5916\times \text{AL}442125\text{.}2\;\text{expression}\;\text{level}\right) \end{array}$$

**TABLE 1:** Nine lncRNAs significantly correlated with the overall survival of hepatocellular carcinoma.

Gene symbol	Ensemble ID	HR	Coefficient	*p*-value
LINC01224	ENSG00000269416	1.9946	0.3222	1.26E-06
C2orf27 A	ENSG00000287151	1.6954	0.0363	1.24E-06
AL158166.1	ENSG00000227076	1.9825	0.2281	7.39E-06
AL117336.2	ENSG00000271335	1.665	0.1265	3.01E-08
MKLN1-AS	ENSG00000236753	3.4874	0.4807	1.36E-09
AL031985.3	ENSG00000260920	1.9409	0.3838	2.27E-11
LINC01138	ENSG00000274020	1.4337	0.0123	3.06E-06
POLH-AS1	ENSG00000203362	2.7838	0.6103	1.29E-06
AL442125.2	ENSG00000276916	3.9656	0.5916	2.79E-05

HR: Hazard ratio.

Patients in the training cohort were assigned to the high- or low-risk group according to the best cutoff value of 2.9, which was determined *via* X-tile software analysis (Figures 4C,D). When analyzing the survival time and status of patients with HCC according to the distribution of risk scores, patients in the high-risk group showed a worse prognosis than those in the low-risk group. The nine lncRNAs were differentially expressed in the high- and low-risk groups, with a higher expression level in the high-risk group (Figures 4E–G). Patients in the validation cohort were also divided into high- and low-risk groups using the same cutoff value of the training cohort (Figures 4H–J). Furthermore, the prognosis of the two groups was compared using the K-M curve, and the results revealed that patients with a high-risk score had a poor prognosis both in the training and validation cohorts (*p* < 0.001, Figures 4K,L). In the training cohort, the 3-year survival rates of the high- and low-risk groups were 31.8 and 72.8%, respectively; in the validation cohort, these rates were 29.9 and 80.2%, respectively.

**FIGURE 5:**
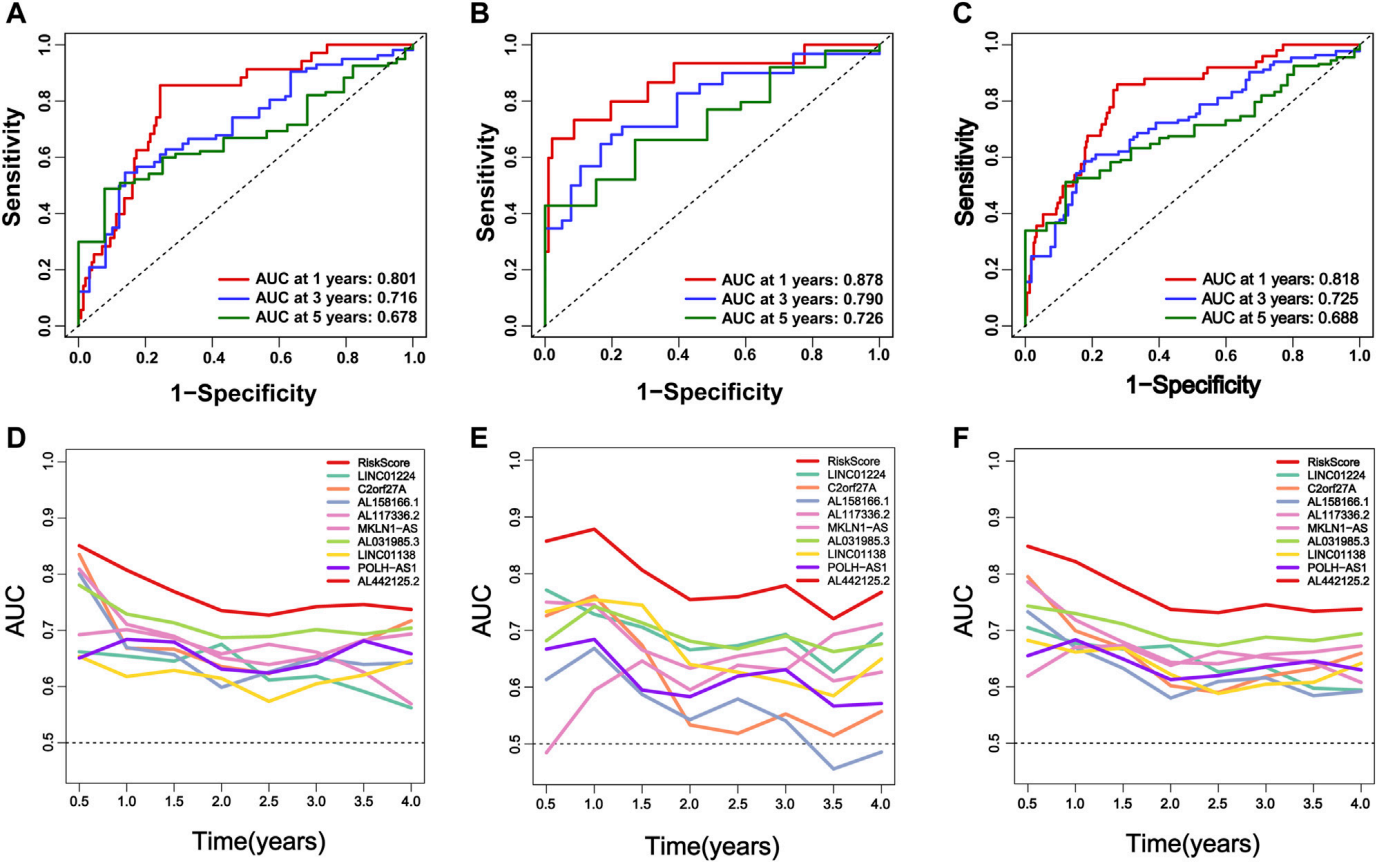
M6A-9LPS predicts the efficiency of OS in patients with HCC in training, validation, and the entire cohort. **(A–C)** The time-dependent ROC curve analysis verified the efficiency of m6A-9LPS in predicting 1-year, 3-year, and 5-year survival rates in the training, validation, and entire TCGA cohort. **(D–F)** The time-dependent AUC value graph compared the predictive efficiency between m6A-9LPS and known prognostic markers in the training cohort, validation, and entire TCGA cohort.

In addition, we evaluated the predictive ability of the established m6A-9LPS signature in the training, validation, and entire cohorts. Regardless of the ROC curve analysis in the three cohorts, m6A-9LPS showed strong predictive capabilities (Figures 5A–C). Moreover, the AUC values of m6A-9LPS were higher than those of single signatures in the training, validation, and entire cohorts, which was confirmed by time-dependent ROC analysis (Figures 5D–F). These results suggest that m6A-9LPS has a strong ability to predict the prognosis of patients with HCC.

### Stratified Survival Analysis of m6A-9LPS in TCGA Cohort

**FIGURE 6:**
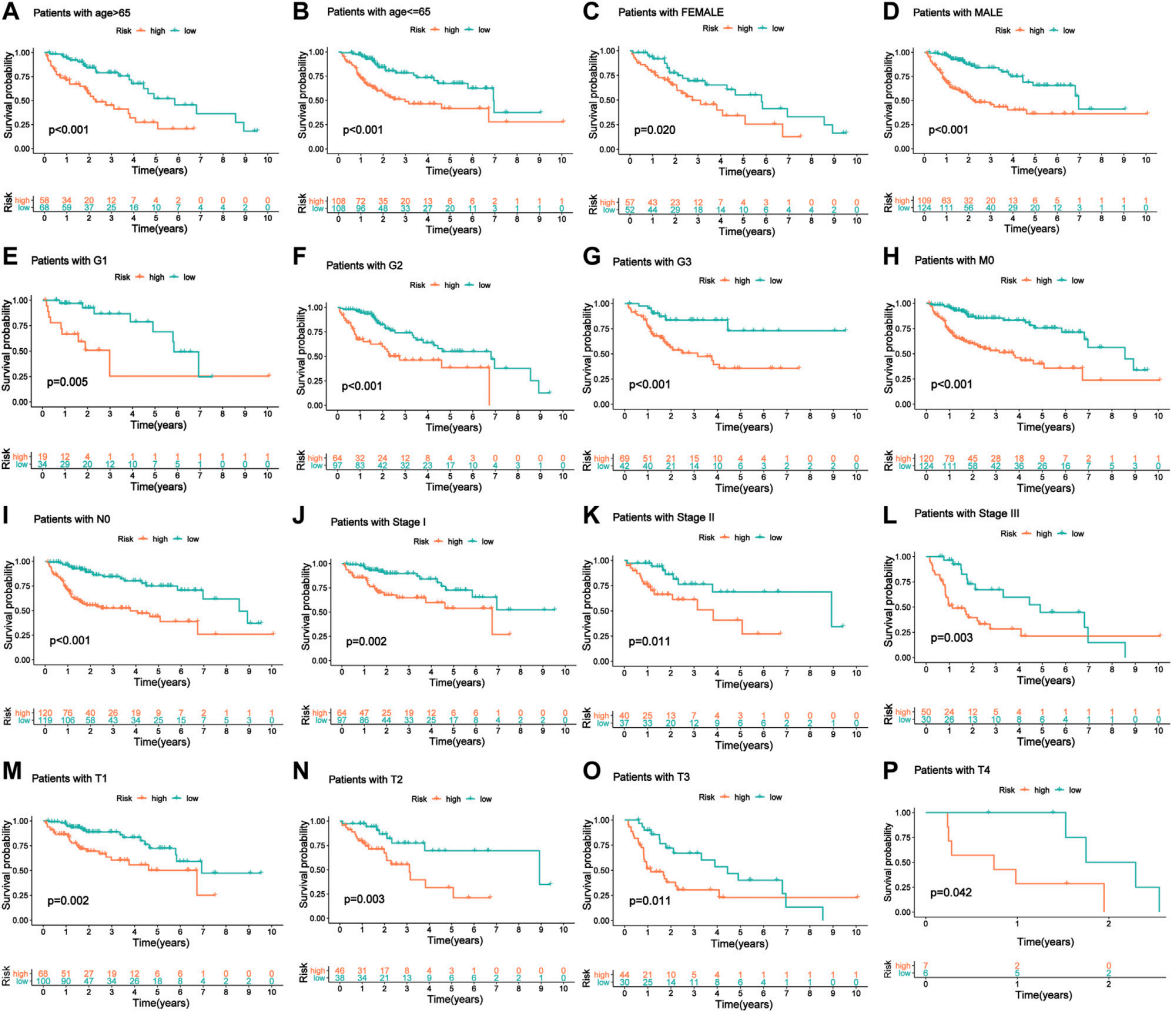
The KM curve of patients with OS stratified by age, gender, and clinical stage according to m6A-9LPS. **(A, B)** KM curve of the elderly and young patients. **(C, D)** KM curve of female and male patients. **(E–G)** KM curve of G1, G2, and G3 stage patients. **(H)** KM curve of patients with the M0 stage group. **(I)** KM curve of patients with the N0 stage group. **(J–L)** KM curve of stage I, stage II, and stage III groups. **(M–P)** KM curves of T1, T2, T3, and T4 stage groups.

To confirm the stability of this model in predicting the prognosis of patients with HCC under different clinicopathological conditions, we performed a stratified analysis on the entire cohort of patients divided into different subgroups based on their different clinical characteristics. Survival analysis revealed significant differences in prognosis between high-risk and low-risk groups classified by the m6A-9LPS model, regardless of sex, age, grade, or AJCC staging. In patients over 65 years of age (*n* = 126) and patients aged 65 or younger (*n* = 216), m6A-9LPS could accurately divide the high- and low-risk groups (*p* < 0.001) (Figures 6A,B); similarly, the male (*n* = 233, *p* < 0.001) and female (*n* = 109, *p* = 0.02) patients were also accurately divided into high- and low-risk groups (Figures 6C,D). In addition, with regard to grade and AJCC staging, the K-M curve showed that the low-risk group had a better OS (Figures 6E–P). However, for patients with grade G4 (*n* = 12, *p* = 0.79), stage N1 (*n* = 3), stage M1 (*n* = 3), and stage IV (*n* = 3), there was no significant difference in prognosis between the high-risk and low-risk groups (Supplementary Figure S2). These results suggest that m6A-9LPS may be a powerful prognostic signature for patients with HCC, and that it is not affected by changes in traditional clinicopathological features.

### Establishment of a Nomogram Combining the m6A-9LPS Signature and Clinicopathological Factors for Predicting the Prognosis of Patients With Hepatocellular Carcinoma

**FIGURE 7:**
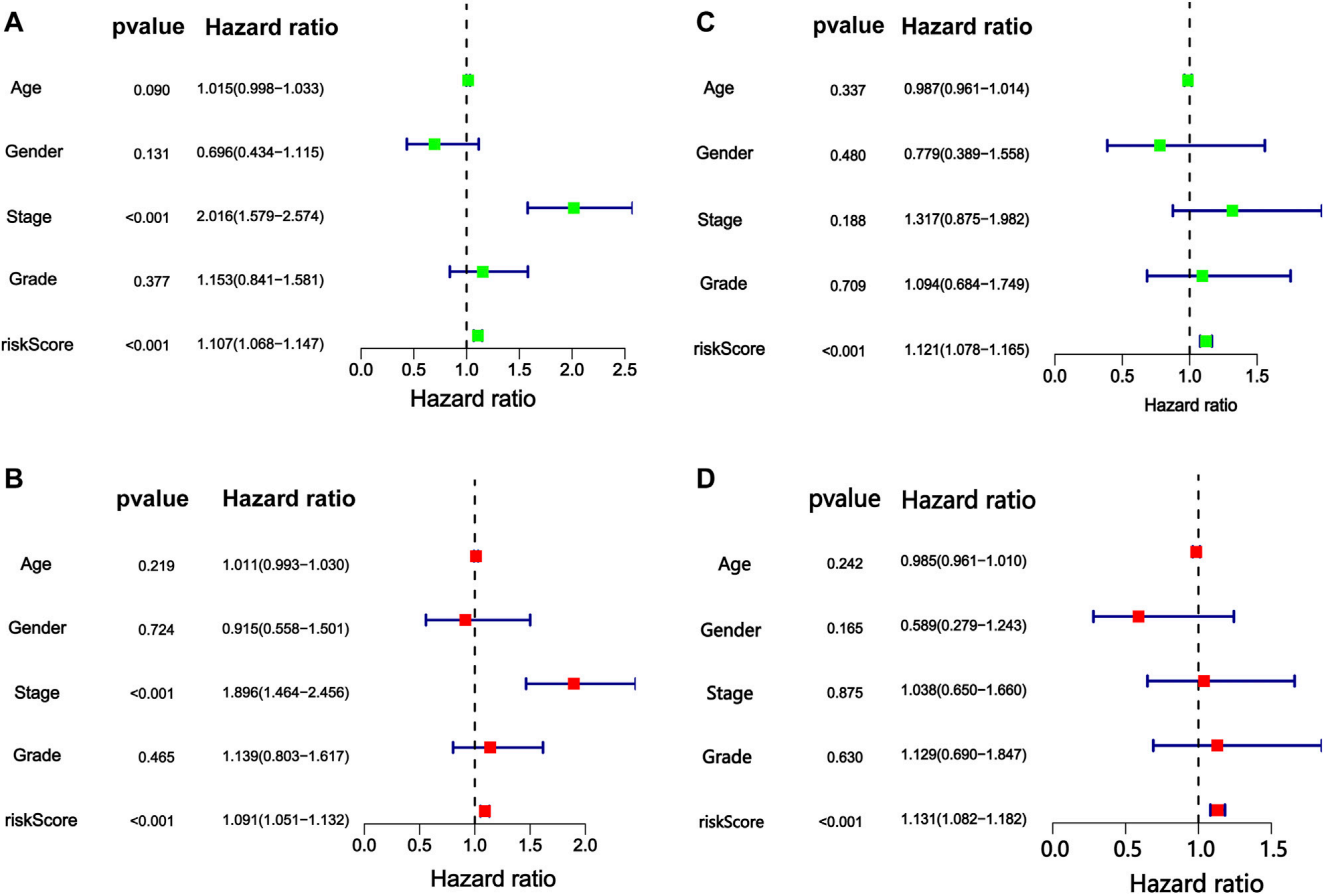
Cox proportional hazards regression analysis of risk score of m6A-9LPS combined with clinical factors. The forest map of **(A)** univariate and **(B)** multivariate Cox regression analysis in the training cohort. The forest map of **(C)** univariate and **(D)** multivariate Cox regression analysis in the validation cohort.

**FIGURE 8:**
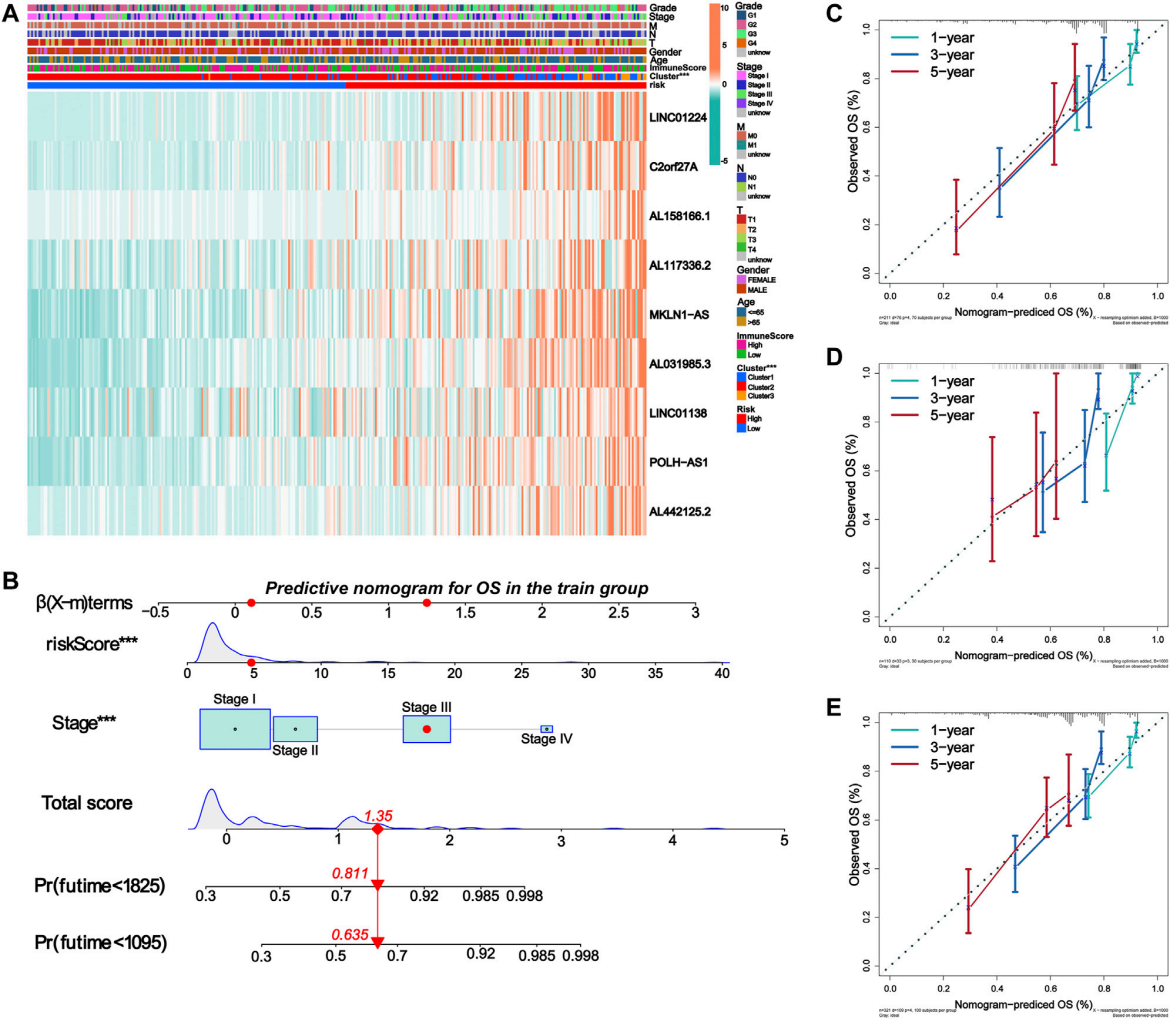
Establishment of nomogram for predicting prognosis of HCC patients. **(A)** The expression of nine lncRNAs in m6A-9LPS was correlated with the clustering characteristics, stromal cell score, immune cell score, comprehensive score, and clinical factors. **(B)** Nomogram for predicting OS of HCC patients constructed based on training cohort patients and significant clinical factors of multivariate Cox regression analysis. **(C–E)** The calibration curves of the Nomogram predicted 1-year, 3-year, and 5-year OS in the training, validation, and entire cohort.

Independent prognostic analysis helped us understand whether m6A-9LPS could be independent of other clinicopathological factors that may affect the prognosis of patients with HCC. Univariate and multivariate Cox regression analyses showed that AJCC staging and m6A-9LPS risk scores were significantly correlated with the prognosis of patients (both *p* < 0.001). This indicates that, the m6A-9LPS risk score is an independent prognostic indicator of HCC (Figures 7A–D). The heatmap shows that the nine lncRNAs were upregulated in the high-risk group. Except for the cluster group (*p* < 0.001), there was no significant difference in the expression of the nine lncRNAs between the high- and low-risk groups (Figure 8A).

We then constructed a nomogram to predict the OS of patients with HCC based on all the significant prognostic factors from the multivariate Cox regression analysis. The nomogram was used to evaluate the 1-year, 3-year, and 5-year OS rates. The total score was calculated based on the scores of all the variables in the nomogram. The OS rate of each patient could be predicted by drawing a vertical line from the total score to the survival prediction axis (Figure 8B). In addition, the C-index of the nomogram was 0.726 (95% CI, 0.663–0.788). The results of the calibration curve analysis showed that, when predicting the OS of patients with HCC at 1 year, 3 years, and 5years, the nomogram had precise predictive ability, and the OS rate was close to reality in the training, validation, and entire cohorts (Figures 8C–E).

### Construction of a ceRNA Network and Enrichment Analysis of Target mRNA Functional Signals

**FIGURE 9:**
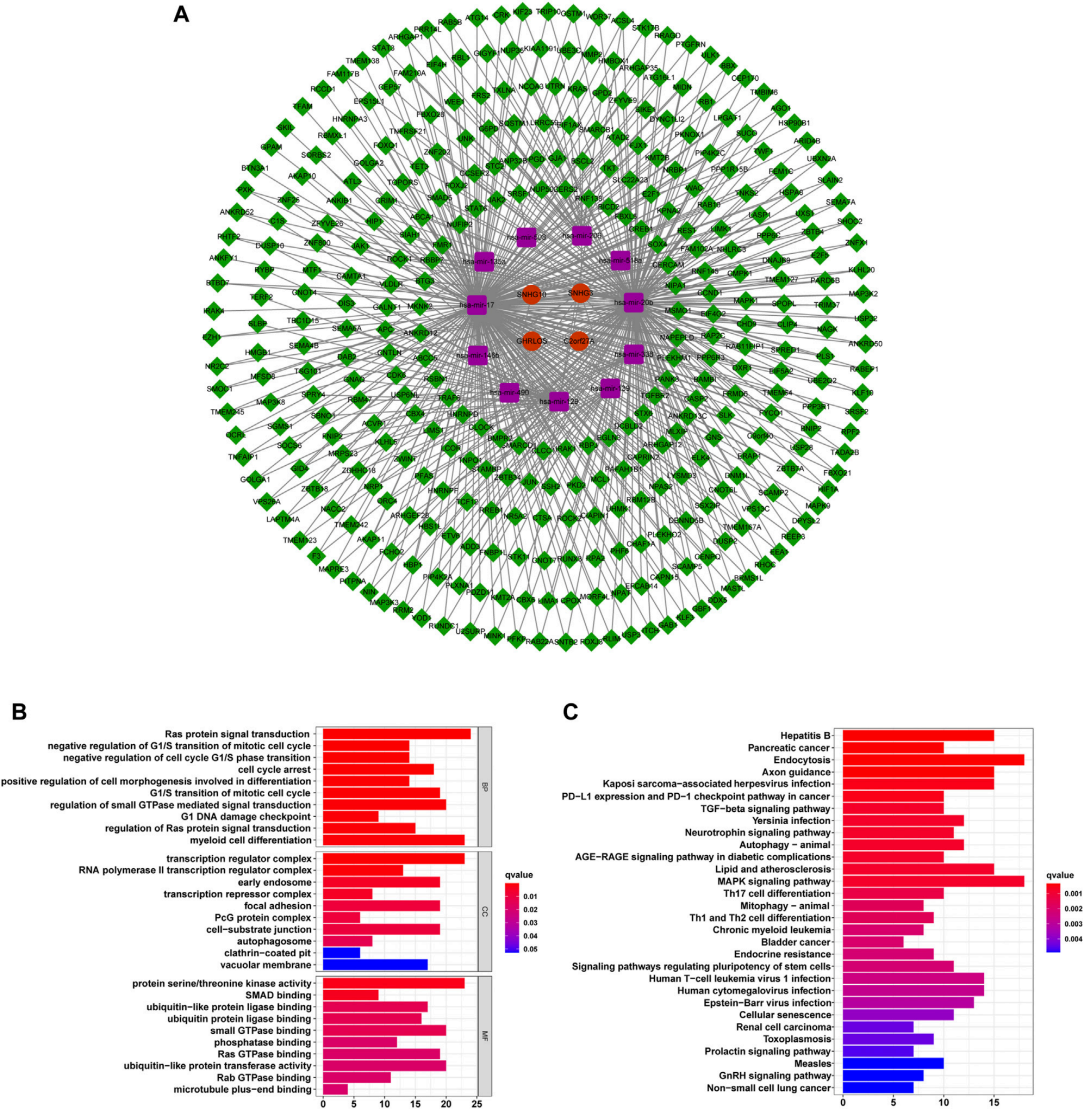
The ceRNA network containing m6ARlncs and their target miRNAs and mRNAs. **(A)** A ceRNA network containing four m6ARlncs (green), 11 miRNAs (purple), and 352 mRNAs (red). Visualization of the results of target mRNA functional signal enrichment, the top five terms of GO **(B)** and the top 30 pathways of KEGG **(C)**.

We constructed a ceRNA network based on prognostic m6ARlncs to illustrate how m6ARlnc can regulate target mRNA expression in HCC *via* sponging miRNA. Four prognostic m6ARlncs were extracted from the miRcode database, and 98 pairs of interactions between the four lncRNAs and 11 miRNAs were identified. Based on the results of 11 miRNAs, 352 target mRNAs were identified. Interestingly, these mRNAs contained the m6A regulator FMR1 (Edupuganti et al., 2017), which confirmed that in HCC, m6ARlnc can act as a ceRNA by targeting m6A regulators through miRNA. Finally, we constructed a ceRNA network of lncRNA–miRNA–mRNA containing four lncRNAs, 11 miRNAs, and 352 mRNAs (Figure 9A). Enrichment analysis of 352 target mRNAs showed that these genes were correlated with enriched Ras protein signal transduction, negative regulation of G1/S transition of mitotic cell cycle, negative regulation of cell cycle G1/S phase transition, cell cycle arrest, and positive regulation of cell morphogenesis involved in differentiation (the top five terms of GO biological processes). The top five KEGG signaling pathways were in hepatitis B, pancreatic cancer, endocytosis, axon guidance, and Kaposi sarcoma-associated herpesvirus infection (Figures 9B,C). This information may provide a reference for further exploration of the function of m6ARlnc in HCC.

### Enrichment Analysis of m6A-9LPS

**FIGURE 10:**
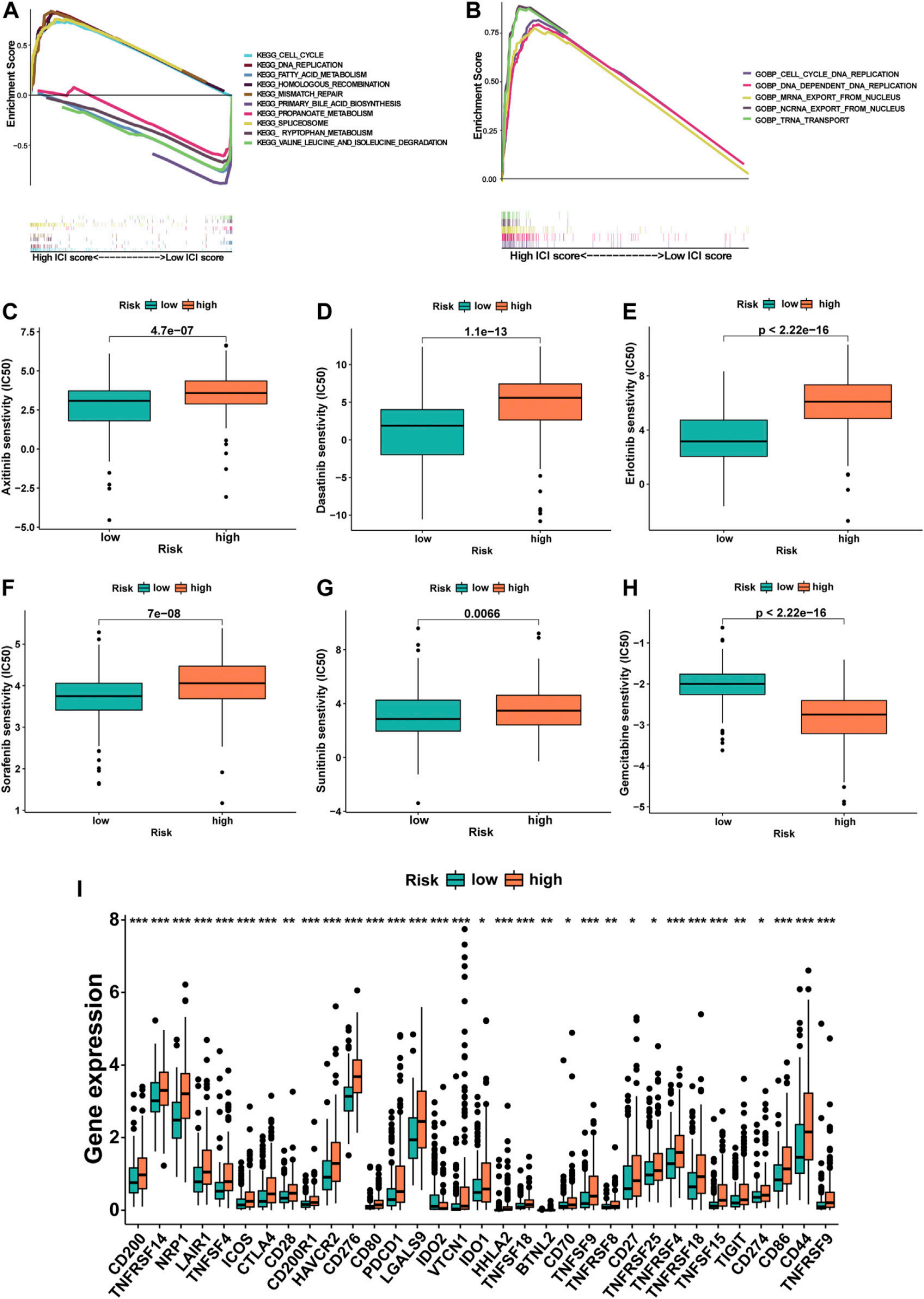
The potential mechanism of m6A-9LPS in TCGA cohort was studied by gene set enrichment analysis. **(A)** Five representative pathways in the high-risk group and five representative pathways in the low-risk group by KEGG enrichment analysis. **(B)** Five representative biological processes in the high-risk group of BP by GO enrichment analysis. The difference of IC50 of axitinib **(C)**, dasatinib **(D)**, erlotinib **(E)**, sorafenib **(F)**, sunitinib **(G)**, and gemcitabine **(H)** between high- and low-risk groups. Box plot of 33 immune checkpoints in entire cohort (green: low-risk group, red: high-risk group; *p*-values were: ***p < 0.001, **p < 0.01, *p < 0.05) **(I)**.

To investigate the biological processes and pathways underlying the molecular heterogeneity between the high- and low-risk m6A-9LPS divided subgroups, we performed KEGG and GO enrichment analyses on the entire cohort. In the GO enrichment analysis, we selected the biological process (BP), combined with the results of the KEGG enrichment analysis, to explore the main biological processes involving lncRNAs in patients with HCC. In the KEGG enrichment analysis, the cell cycle, DNA replication, fatty acid metabolism, homologous recombination, and mismatch repair pathways were significantly enriched in the tumors of the m6A-9LPS-defined high-risk group. In the low-risk group, the significantly enriched pathways were mainly related to various metabolic processes (including primary bile acid biosynthesis, tryptophan metabolism, and acetone metabolism), spliceosomes, and the degradation of certain amino acids (valine, leucine, and isoleucine) (Figure 10A). The results of GO enrichment analysis showed that in the high-risk group, cell cycle DNA replication, DNA-dependent replication, mRNA export from the nucleus, ncRNA export from the nucleus, and tRNA transport played important roles, while no significant biological processes were enriched in the low-risk group (Figure 10B).

### Responses of Patients to Chemotherapy and Immunotherapy Based on the Classification of m6A-9LPS

The IC50 of six commonly used chemotherapeutic agents was predicted in high- and low-risk groups for HCC, which was based on the algorithm provided in the *pRRophetic* R package. The IC50 values of axitinib, dasatinib, erlotinib, sorafenib, and sunitinib were higher in the high-risk group, indicating that patients in the low-risk group were more sensitive to these five drugs; at the same time, the IC50 of gemcitabine was higher in low-risk patients, which meant that high-risk patients were more sensitive to gemcitabine (Figures 10C–H). In addition, the relationship between the gene expression levels of immune checkpoints and the risk group was further analyzed. The expression levels of 33 immune checkpoints in the high-risk group were significantly higher than those in the low-risk group (*p* < 0.001, Figure 10I).

## Discussion

The change in lncRNAs can be used as a target or biomarker for disease diagnosis and stage definition. Many studies have reported that lncRNAs play an essential role in the invasion, migration, proliferation, apoptosis, and drug resistance of a variety of tumors (Wei and Wang 2017; Zhao et al., 2018; Luo et al., 2019; Zhuang et al., 2019; Shi et al., 2020). More importantly, lncRNAs are involved in the occurrence and development of HCC (Wong et al., 2018; Huang et al., 2020), with m6A modification strongly regulating the RNA life cycle. The m6A modification in tumors is primarily encoded by “writers” that catalyze the formation of m6A (such as METL16, RBM15, and ZC3H13), “erasers” that selectively remove m6A (FTO and ALKBH5), and “readers” that decode the methylation of m6A (such as YTHDC1, FMR1, and RBMX) (Jung and Goldman 2018; Meng et al., 2020; Wang et al., 2020). m6A has been shown to be involved in the progression of various cancers (Alarcon et al., 2015; Zhao et al., 2017; Liu J. et al., 2019), but there are few studies on the regulation of m6A regulators on lncRNAs in tumors. Our study attempted to focus on the relationship between m6A regulator-related lncRNAs and their association with the prognosis of patients with HCC, aiming to establish a new prediction signature of m6ARlnc, explore its potential underlying molecular mechanisms, and evaluate its clinical application in foreseeing the prognosis of patients and determining the most suitable treatment.

In the entire cohort, the results of the co-expression network suggested that many lncRNAs were related to m6A regulators, which stimulated our interest in exploring the expression and role of m6ARlncs in HCC. At the same time, we further explored the relationship between the subgroups enriched with prognostic m6ARlnc and the OS, tumor cell immune infiltration, and TME of patients through consensus clustering analysis. The results suggested that prognostic m6ARlncs have a potential predictive effect. CIBERSORT analysis showed that regulatory T cells and naïve B cells were highly infiltrated in Cluster2; in the results provided by the ESTIMATE algorithm, stromal cell score of Cluster2 was higher. Therefore, the activated regulatory T cells and naïve B cells infiltration in the TME may be related to the poor prognosis of HCC, which is consistent with the results of previous studies on immunity in other tumors (Ma C. et al., 2020; Song and Wu 2020), providing novel insights for patients with HCC on immunotherapy choice.

A total of 342 patients from the TCGA dataset were randomly divided into training and validation cohorts to explore the prognostic value of m6ARlnc. Finally, nine lncRNAs were selected to establish the risk signature, and survival analysis showed that our model could strongly predict the OS of patients in the training, validation, and entire cohorts. To the best of our knowledge, lncRNA-regulated miRNA, chemotherapeutic agent sensitivity, lncRNA mutation, and immunotherapy are closely related to tumor progression. LINC01224, LINC01138, and MKLN1-AS affect the progression of HCC by regulating miRNAs; the upregulation of C2orf27 A was found to be related to resistance to sorafenib in HCC; POLH-AS1 mutation is associated with the occurrence and development of many diseases, but it has not been reported in HCC (Flanagan et al., 2007; Jiang et al., 2019; Gong D. et al., 2020; Gao et al., 2020; Kong et al., 2020; Yuan et al., 2021). Thus, we can say that the lncRNAs in m6A-9LPS are closely related to the progression of cancer and its therapy. In addition, three of these lncRNAs have never been reported; hence, we speculate that further research may be helpful for the early diagnosis of HCC and the development of new prognostic signatures.

Additionally, a nomogram was constructed and validated. The c-index of the nomogram and the 1-year, 3-year, and 5-year calibration curves of patients with HCC showed that the nomogram prediction was highly consistent with the actual survival. Additionally, in the validation and entire cohorts, the nomogram showed excellent predictive ability, providing clinicians with an easy-to-develop prognosis prediction tool.

Through KEGG and GO enrichment analyses, we revealed the potential biological processes and pathways underlying m6A-9LPS. It is worth noting that the scores of patients in the high-risk group were closely related to the cell cycle, DNA replication, and RNA transport. The cell cycle is a regulatory process closely associated with cell reproductive capacity, and its repetition leads to exponential cell proliferation (Coffman 2004). An imbalance in the cell cycle is very common in tumors, and its expression can result in abnormal proliferation or apoptosis (Evan and Vousden 2001). DNA replication is a critical process in cell proliferation, and many studies have shown that it is an important hallmark of cancer (Evan and Vousden 2001; Masai et al., 2010). RNA modification can affect RNA transport through RNA interference (Witkin et al., 2015); therefore, we speculate that the type of RNA modification involved in HCC may be m6A, confirming the rationale of our study. The metabolism-related pathways and amino acid degradation were more prominent in the low-risk group. Metabolic reprogramming plays an important role in tumor cells, which, in the presence of abundant nutrients, can promote the transformation of carbon into biomacromolecules *via* the activation of metabolic pathways (Boroughs and DeBerardinis 2015). At the same time, the imbalance of amino acid degradation also affects the state of tumor cells and the metabolism of patients (Sivanand and Vander Heiden, 2020). In addition, m6ARlncs may play the role of ce-RNA, targeting m6A regulators to affect the occurrence and development of HCC. At the same time, the results of target mRNA enrichment of m6ARlncs were similar to those of the previous enrichment analyses of patients in the high- and low-risk groups, mainly related to the cell cycle and certain malignant processes. In summary, m6A-9LPS may mediate the occurrence and development of HCC through the above-mentioned pathways; however, further experimental research is needed to explain the potential role of these lncRNAs in HCC. Moreover, in the follow-up research, more attention should be paid to the interaction between lncRNAs and m6A regulators, in order to gain a deeper understanding of the potential mechanisms underlying the occurrence and development of HCC.

It is well known that HCC is resistant to chemotherapeutic drugs; hence, it is very important to choose individualized chemotherapeutic agents (Feng et al., 2020; Xu et al., 2020). The predictive results on the sensitivity to six chemotherapeutic agents suggested that gemcitabine is an ideal choice for patients with HCC in the high-risk group, whereas axitinib, dasatinib, erlotinib, sorafenib, and sunitinib may have a better effect in patients in the low-risk group. However, further experiments are necessary to validate these results.

Immunotherapy has become a promising method to cure HCC, according to emerging studies (Liu Z. et al., 2019). The immune checkpoints play an indispensable role in tumors in maintaining self-tolerance and regulating the duration and intensity of the immune response. Blocking the immune checkpoints has become a new method for eliminating the immunosuppressive microenvironment, thus achieving tumor immunotherapy (Ribas and Wolchok 2018). Our results showed that the expression levels of 33 immune checkpoints were significantly different between the m6A-9LPS-defined high- and low-risk groups, with an upregulation in the high-risk group. The use of immunosuppressive agents in high-risk patients may be effective.

However, our study had some limitations. First, this was a retrospective analysis based on open-source databases, so to avoid the possible deviation of the data from different platforms, we used the internal validation method in the TCGA cohort; if possible, it should also be validated in other independent cohorts. Additionally, the potential mechanism of m6A-9LPS may need to be further validated through *in vitro* and *in vivo* studies.

## Data Availability

The original contributions presented in the study are included in the article/Supplementary Material. Further inquiries can be directed to the corresponding authors.

## References

[B1] Agris P. F. Narendran A. Sarachan K. Väre V. Y. P. Eruysal E. (2017). The Importance of Being Modified. Enzymes 41, 1–50. 10.1016/bs.enz.2017.03.005 28601219 PMC8118379

[B2] Alarcón C. R. Lee H. Goodarzi H. Halberg N. Tavazoie S. F. (2015). N6-methyladenosine marks Primary microRNAs for Processing. Nature 519 (7544), 482–485. 10.1038/nature14281 25799998 PMC4475635

[B3] Boroughs L. K. DeBerardinis R. J. (2015). Metabolic Pathways Promoting Cancer Cell Survival and Growth. Nat. Cel Biol. 17 (4), 351–359. 10.1038/ncb3124 PMC4939711 25774832

[B4] Cao Y.-W. Li W.-Q. Wan G.-X. Li Y.-X. Du X.-M. Li Y.-C. (2014). Correlation and Prognostic Value of SIRT1 and Notch1 Signaling in Breast Cancer. J. Exp. Clin. Cancer Res. 33, 97. 10.1186/s13046-014-0097-2 25420528 PMC4248440

[B5] Chen S. Zhou L. Wang Y. (2020a). ALKBH5-mediated m6A Demethylation of lncRNA PVT1 Plays an Oncogenic Role in Osteosarcoma. Cancer Cel Int. 20, 34. 10.1186/s12935-020-1105-6 PMC6993345 32021563

[B6] Chen Y. Bao C. Zhang X. Lin X. Fu Y. (2020b). Knockdown of LINC00662 Represses AK4 and Attenuates Radioresistance of Oral Squamous Cell Carcinoma. Cancer Cel Int. 20, 244. 10.1186/s12935-020-01286-9 PMC7296632 32549791

[B7] Coffman J. A. (2004). Cell Cycle Development. Develop. Cel 6 (3), 321–327. 10.1016/s1534-5807(04)00067-x 15030756

[B8] Dong Z. Cui H. (2020). The Emerging Roles of RNA Modifications in Glioblastoma. Cancers 12 (3), 736. 10.3390/cancers12030736 PMC7140112 32244981

[B9] Edupuganti R. R. Geiger S. Lindeboom R. G. H. Shi H. Hsu P. J. Lu Z. (2017). N6-methyladenosine (m6A) Recruits and Repels Proteins to Regulate mRNA Homeostasis. Nat. Struct. Mol. Biol. 24 (10), 870–878. 10.1038/nsmb.3462 28869609 PMC5725193

[B10] Evan G. I. Vousden K. H. (2001). Proliferation, Cell Cycle and Apoptosis in Cancer. Nature 411 (6835), 342–348. 10.1038/35077213 11357141

[B11] Feng J. Dai W. Mao Y. Wu L. Li J. Chen K. (2020). Simvastatin Re-sensitizes Hepatocellular Carcinoma Cells to Sorafenib by Inhibiting HIF-1α/PPAR-γ/PKM2-Mediated Glycolysis. J. Exp. Clin. Cancer Res. 39 (1), 24. 10.1186/s13046-020-1528-x 32000827 PMC6993409

[B12] Flanagan A. Rafferty G. O'Neill A. Rynne L. Kelly J. McCann J. (2007). The Human POLH Gene Is Not Mutated, and Is Expressed in a Cohort of Patients with Basal or Squamous Cell Carcinoma of the Skin. Int. J. Mol. Med. 19 (4), 589–596. 10.3892/ijmm.19.4.589 17334634

[B13] Gao W. Chen X. Chi W. Xue M. (2020). Long Noncoding RNA MKLN1AS Aggravates Hepatocellular Carcinoma Progression by Functioning as a Molecular Sponge for miR6543p, Thereby Promoting Hepatoma Derived Growth Factor Expression. Int. J. Mol. Med. 46 (5), 1743–1754. 10.3892/ijmm.2020.4722 33000222 PMC7521589

[B14] Geeleher P. Cox N. Huang R. S. (2014). pRRophetic: an R Package for Prediction of Clinical Chemotherapeutic Response from Tumor Gene Expression Levels. PLoS One 9 (9), e107468. 10.1371/journal.pone.0107468 25229481 PMC4167990

[B15] Gong D. Feng P.-C. Ke X.-F. Kuang H.-L. Pan L.-L. Ye Q. (2020a). Silencing Long Non-coding RNA LINC01224 Inhibits Hepatocellular Carcinoma Progression via MicroRNA-330-5p-Induced Inhibition of CHEK1. Mol. Ther. Nucleic Acids 19, 482–497. 10.1016/j.omtn.2019.10.007 31902747 PMC6948252

[B16] Gong J. Wang Y. Shu C. (2020b). LncRNA CHRF Promotes Cell Invasion and Migration via EMT in Gastric Cancer. Eur. Rev. Med. Pharmacol. Sci. 24 (3), 1168–1176. 10.26355/eurrev_202002_20168 32096147

[B17] Hong W. Liang L. Gu Y. Qi Z. Qiu H. Yang X. (2020). Immune-Related lncRNA to Construct Novel Signature and Predict the Immune Landscape of Human Hepatocellular Carcinoma. Mol. Ther. Nucleic Acids 22, 937–947. 10.1016/j.omtn.2020.10.002 33251044 PMC7670249

[B18] Hou J. Wang Z. Li H. Zhang H. Luo L. (2020). Gene Signature and Identification of Clinical Trait-Related M6 A Regulators in Pancreatic Cancer. Front. Genet. 11, 522. 10.3389/fgene.2020.00522 32754191 PMC7367043

[B19] Huang T. Gao Q. Feng T. Zheng Y. Guo J. Zeng W. (2018). FTO Knockout Causes Chromosome Instability and G2/M Arrest in Mouse GC-1 Cells. Front. Genet. 9, 732. 10.3389/fgene.2018.00732 30719031 PMC6348250

[B20] Huang Z. Zhou J.-K. Peng Y. He W. Huang C. (2020). The Role of Long Noncoding RNAs in Hepatocellular Carcinoma. Mol. Cancer 19 (1), 77. 10.1186/s12943-020-01188-4 32295598 PMC7161154

[B21] Jiang H. Shi X. Ye G. Xu Y. Xu J. Lu J. (2019). Up-regulated Long Non-coding RNA DUXAP8 Promotes Cell Growth through Repressing Krüppel-like Factor 2 Expression in Human Hepatocellular Carcinoma. Onco. Targets Ther. 12, 7429–7436. 10.2147/OTT.S214336 31571902 PMC6750713

[B22] Jung H. I. Jeong D. Ji S. Ahn T. S. Bae S. H. Chin S. (2017). Overexpression of PD-L1 and PD-L2 Is Associated with Poor Prognosis in Patients with Hepatocellular Carcinoma. Cancer Res. Treat. 49 (1), 246–254. 10.4143/crt.2016.066 27456947 PMC5266389

[B23] Jung Y. Goldman D. (2018). Role of RNA Modifications in Brain and Behavior. Genes Brain Behav. 17 (3), e12444. 10.1111/gbb.12444 29244246 PMC6233296

[B24] Kong W. Wang X. Zuo X. Mao Z. Cheng Y. Chen W. (2020). Development and Validation of an Immune-Related lncRNA Signature for Predicting the Prognosis of Hepatocellular Carcinoma. Front. Genet. 11, 1037. 10.3389/fgene.2020.01037 33101369 PMC7500314

[B25] Kopp F. Mendell J. T. (2018). Functional Classification and Experimental Dissection of Long Noncoding RNAs. Cell 172 (3), 393–407. 10.1016/j.cell.2018.01.011 29373828 PMC5978744

[B26] Kung J. T. Y. Lee J. T. (2013). RNA in the Loop. Develop. Cel 24 (6), 565–567. 10.1016/j.devcel.2013.03.009 PMC3777248 23537627

[B27] Li B. Mao R. Liu C. Zhang W. Tang Y. Guo Z. (2018). LncRNA FAL1 Promotes Cell Proliferation and Migration by Acting as a CeRNA of miR-1236 in Hepatocellular Carcinoma Cells. Life Sci. 197, 122–129. 10.1016/j.lfs.2018.02.006 29421439

[B28] Lin Z. Ni X. Dai S. Chen H. Chen J. Wu B. (2020). Screening and Verification of Long Noncoding RNA Promoter Methylation Sites in Hepatocellular Carcinoma. Cancer Cel Int. 20, 311. 10.1186/s12935-020-01407-4 PMC7362420 32684848

[B29] Liu J. Harada B. T. He C. (2019a). Regulation of Gene Expression by N-Methyladenosine in Cancer. Trends Cel Biol. 29 (6), 487–499. 10.1016/j.tcb.2019.02.008 PMC6527461 30940398

[B30] Liu Z. Lin Y. Zhang J. Zhang Y. Li Y. Liu Z. (2019b). Molecular Targeted and Immune Checkpoint Therapy for Advanced Hepatocellular Carcinoma. J. Exp. Clin. Cancer Res. 38 (1), 447. 10.1186/s13046-019-1412-8 31684985 PMC6827249

[B31] Luo H. Xu C. Le W. Ge B. Wang T. (2019). lncRNA CASC11 Promotes Cancer Cell Proliferation in Bladder Cancer through miRNA‐150. J. Cel Biochem. 120 (8), 13487–13493. 10.1002/jcb.28622 PMC6619255 30916832

[B32] Ma C. Luo H. Cao J. Zheng X. Zhang J. Zhang Y. (2020a). Identification of a Novel Tumor Microenvironment-Associated Eight-Gene Signature for Prognosis Prediction in Lung Adenocarcinoma. Front. Mol. Biosci. 7, 571641. 10.3389/fmolb.2020.571641 33102522 PMC7546815

[B33] Ma X. Li Y. Wen J. Zhao Y. (2020b). m6A RNA Methylation Regulators Contribute to Malignant Development and Have a Clinical Prognostic Effect on Cervical Cancer. Am. J. Transl. Res. 12 (12), 8137–8146. 33437387 PMC7791487

[B34] Masai H. Matsumoto S. You Z. Yoshizawa-Sugata N. Oda M. (2010). Eukaryotic Chromosome DNA Replication: where, when, and How? Annu. Rev. Biochem. 79, 89–130. 10.1146/annurev.biochem.052308.103205 20373915

[B35] Meng Z. Yuan Q. Zhao J. Wang B. Li S. Offringa R. (2020). The m6A-Related mRNA Signature Predicts the Prognosis of Pancreatic Cancer Patients. Mol. Ther. Oncolytics 17, 460–470. 10.1016/j.omto.2020.04.011 32490170 PMC7256444

[B36] Meyer K. D. Jaffrey S. R. (2017). Rethinking m6A Readers, Writers, and Erasers. Annu. Rev. Cel Dev. Biol. 33, 319–342. 10.1146/annurev-cellbio-100616-060758 PMC5963928 28759256

[B37] Pan W. Li W. Zhao J. Huang Z. Zhao J. Chen S. (2019). Lnc RNA ‐ PDPK 2P Promotes Hepatocellular Carcinoma Progression through the PDK 1/AKT/Caspase 3 Pathway. Mol. Oncol. 13 (10), 2246–2258. 10.1002/1878-0261.12553 31368655 PMC6763783

[B38] Ribas A. Wolchok J. D. (2018). Cancer Immunotherapy Using Checkpoint Blockade. Science 359 (6382), 1350–1355. 10.1126/science.aar4060 29567705 PMC7391259

[B39] Robinson M. Shah P. Cui Y.-H. He Y.-Y. (2019). The Role of Dynamic m6A RNA Methylation in Photobiology. Photochem. Photobiol. 95 (1), 95–104. 10.1111/php.12930 29729018 PMC6309319

[B40] Roundtree I. A. Evans M. E. Pan T. He C. (2017). Dynamic RNA Modifications in Gene Expression Regulation. Cell 169 (7), 1187–1200. 10.1016/j.cell.2017.05.045 28622506 PMC5657247

[B41] Shi Q. Li Y. Li S. Jin L. Lai H. Wu Y. (2020). LncRNA DILA1 Inhibits Cyclin D1 Degradation and Contributes to Tamoxifen Resistance in Breast Cancer. Nat. Commun. 11 (1), 5513. 10.1038/s41467-020-19349-w 33139730 PMC7608661

[B42] Siegel R. L. Miller K. D. Jemal A. (2020). Cancer Statistics, 2020. CA A. Cancer J. Clin. 70 (1), 7–30. 10.3322/caac.21590 31912902

[B43] Sivanand S. Vander Heiden M. G. (2020). Emerging Roles for Branched-Chain Amino Acid Metabolism in Cancer. Cancer Cell 37 (2), 147–156. 10.1016/j.ccell.2019.12.011 32049045 PMC7082774

[B44] Song J. Wu L. (2020). Friend or Foe: Prognostic and Immunotherapy Roles of BTLA in Colorectal Cancer. Front. Mol. Biosci. 7, 148. 10.3389/fmolb.2020.00148 32793631 PMC7385242

[B45] Tran N. T. Su H. Khodadadi‐Jamayran A. Lin S. Zhang L. Zhou D. (2016). The AS‐RBM15 lncRNA Enhances RBM15 Protein Translation during Megakaryocyte Differentiation. EMBO Rep. 17 (6), 887–900. 10.15252/embr.201541970 27118388 PMC5278610

[B46] Wang H. Zhao X. Lu Z. (2021). m6A RNA Methylation Regulators Act as Potential Prognostic Biomarkers in Lung AdenocarcinomaA RNA Methylation Regulators Act as Potential Prognostic Biomarkers in Lung Adenocarcinoma. Front. Genet. 12, 622233. 10.3389/fgene.2021.622233 33643384 PMC7902930

[B47] Wang T. Kong S. Tao M. Ju S. (2020). The Potential Role of RNA N6-Methyladenosine in Cancer Progression. Mol. Cancer 19 (1), 88. 10.1186/s12943-020-01204-7 32398132 PMC7216508

[B48] Wei G. H. Wang X. (2017). lncRNA MEG3 Inhibit Proliferation and Metastasis of Gastric Cancer via P53 Signaling Pathway. Eur. Rev. Med. Pharmacol. Sci. 21 (17), 3850–3856. 28975980

[B49] Witkin K. L. Hanlon S. E. Strasburger J. A. Coffin J. M. Jaffrey S. R. Howcroft T. K. (2015). RNA Editing, Epitranscriptomics, and Processing in Cancer Progression. Cancer Biol. Ther. 16 (1), 21–27. 10.4161/15384047.2014.987555 25455629 PMC4622672

[B50] Wong C.-M. Tsang F. H.-C. Ng I. O.-L. (2018). Non-coding RNAs in Hepatocellular Carcinoma: Molecular Functions and Pathological Implications. Nat. Rev. Gastroenterol. Hepatol. 15 (3), 137–151. 10.1038/nrgastro.2017.169 29317776

[B51] Wu X. Zhang X. Tao L. Dai X. Chen P. (2020). Prognostic Value of an m6A RNA Methylation Regulator-Based Signature in Patients with Hepatocellular Carcinoma. Biomed. Res. Int. 2020, 1–11. 10.1155/2020/2053902 PMC7378627 32733931

[B52] Xu W.-P. Liu J.-P. Feng J.-F. Zhu C.-P. Yang Y. Zhou W.-P. (2020). miR-541 Potentiates the Response of Human Hepatocellular Carcinoma to Sorafenib Treatment by Inhibiting Autophagy. Gut 69 (7), 1309–1321. 10.1136/gutjnl-2019-318830 31727683

[B53] Yoo J.-J. Chung G. E. Lee J.-H. Nam J. Y. Chang Y. Lee J. M. (2018). Sub-classification of Advanced-Stage Hepatocellular Carcinoma: A Cohort Study Including 612 Patients Treated with Sorafenib. Cancer Res. Treat. 50 (2), 366–373. 10.4143/crt.2017.126 28521494 PMC5912123

[B54] Yuan W. Tao R. Huang D. Yan W. Shen G. Ning Q. (2021). Transcriptomic Characterization Reveals Prognostic Molecular Signatures of Sorafenib Resistance in Hepatocellular Carcinoma. Aging 13 (3), 3969–3993. 10.18632/aging.202365 33495404 PMC7906139

[B55] Yuan Y. Du Y. Wang L. Liu X. (2020). The M6A Methyltransferase METTL3 Promotes the Development and Progression of Prostate Carcinoma via Mediating MYC Methylation. J. Cancer 11 (12), 3588–3595. 10.7150/jca.42338 32284755 PMC7150444

[B56] Zhang B. Gu Y. Jiang G. (2020a). Expression and Prognostic Characteristics of M6 A RNA Methylation Regulators in Breast Cancer. Front. Genet. 11, 604597. 10.3389/fgene.2020.604597 33362863 PMC7758326

[B57] Zhang S. Liu F. Wu Z. Xie J. Yang Y. Qiu H. (2020b). Contribution of m6A Subtype Classification on Heterogeneity of Sepsis. Ann. Transl. Med. 8 (6), 306. 10.21037/atm.2020.03.07 32355750 PMC7186660

[B58] Zhang Y. X. Yuan J. Gao Z. M. Zhang Z. G. (2018). LncRNA TUC338 Promotes Invasion of Lung Cancer by Activating MAPK Pathway. Eur. Rev. Med. Pharmacol. Sci. 22 (2), 443–449. 10.26355/eurrev_201801_14193 29424901

[B59] Zhao B. S. Roundtree I. A. He C. (2017). Post-transcriptional Gene Regulation by mRNA Modifications. Nat. Rev. Mol. Cel Biol. 18 (1), 31–42. 10.1038/nrm.2016.132 PMC5167638 27808276

[B60] Zhao W. Geng D. Li S. Chen Z. Sun M. (2018). LncRNA HOTAIR Influences Cell Growth, Migration, Invasion, and Apoptosis via the miR-20a-5p/HMGA2axis in Breast Cancer. Cancer Med. 7 (3), 842–855. 10.1002/cam4.1353 29473328 PMC5852357

[B61] Zhao Z. Yang L. Fang S. Zheng L. Wu F. Chen W. (2020). The Effect of m6A Methylation Regulatory Factors on the Malignant Progression and Clinical Prognosis of Hepatocellular Carcinoma. Front. Oncol. 10, 1435. 10.3389/fonc.2020.01435 32974160 PMC7471744

[B62] Zhu L. Zhu Y. Han S. Chen M. Song P. Dai D. (2019). Impaired Autophagic Degradation of lncRNA ARHGAP5-AS1 Promotes Chemoresistance in Gastric Cancer. Cell Death Dis. 10 (6), 383. 10.1038/s41419-019-1585-2 31097692 PMC6522595

[B63] Zhuang C. Ma Q. Zhuang C. Ye J. Zhang F. Gui Y. (2019). LncRNA GClnc1 Promotes Proliferation and Invasion of Bladder Cancer through Activation of MYC. FASEB J. 33 (10), 11045–11059. 10.1096/fj.201900078RR 31298933

