# Supplementary material for: Identification and Validation of a Prognostic Prediction Model of m6A Regulator-Related LncRNAs in Hepatocellular Carcinoma
Source: Front Mol Biosci. 2021 Dec 20;8:784553. doi: 10.3389/fmolb.2021.784553 (PMC8721125; doi:10.3389/fmolb.2021.784553)
Supplement: Supplementary file 1 [file DataSheet2.docx]

**SUPPLEMENTARY TABLE 1 |** Details of twenty-three m6A regulators.

| **Gene symbol** | **Full name** | **Type** |
| --- | --- | --- |
| METTL3 | methyltransferase 3 | Writers |
| METTL14 | methyltransferase 14 | Writers |
| METTL16 | methyltransferase 16 | Writers |
| WTAP | WT1 associated protein | Writers |
| VIRMA | vir like m6A methyltransferase associated | Writers |
| ZC3H13 | zinc finger CCCH-type containing 13 | Writers |
| RBM15 | RNA binding motif protein 15 | Writers |
| RBM15B | RNA binding motif protein 15B | Writers |
| YTHDC1 | YTH domain containing 1 | Readers |
| YTHDC2 | YTH domain containing 2 | Readers |
| YTHDF1 | YTH N6-methyladenosine RNA binding protein 1 | Readers |
| YTHDF2 | YTH N6-methyladenosine RNA binding protein 2 | Readers |
| YTHDF3 | YTH N6-methyladenosine RNA binding protein 3 | Readers |
| HNRNPC | heterogeneous nuclear ribonucleoprotein C | Readers |
| FMR1 | FMRP translational regulator 1 | Readers |
| LRPPRC | leucine rich pentatricopeptide repeat containing | Readers |
| HNRNPA2B1 | heterogeneous nuclear ribonucleoprotein A2/B1 | Readers |
| IGF2BP1 | insulin like growth factor 2 mRNA binding protein 1 | Readers |
| IGF2BP2 | insulin like growth factor 2 mRNA binding protein 2 | Readers |
| IGF2BP3 | insulin like growth factor 2 mRNA binding protein 3 | Readers |
| RBMX | RNA binding motif protein X-linked | Readers |
| FTO | FTO alpha-ketoglutarate dependent dioxygenase | Erasers |
| ALKBH5 | alkB homolog 5, RNA demethylase | Erasers |

**SUPPLEMENTARY TABLE 2 |** Details of thirty-three immune checkpoints used to evaluate m6A-9LPS for immunotherapy in HCC patients.

| **Gene symbol** | **Full name** | **Ensemble ID** |
| --- | --- | --- |
| IDO1 | indoleamine 2,3-dioxygenase 1 | ENSG00000131203 |
| CTLA4 | cytotoxic T-lymphocyte associated protein 4 | ENSG00000163599 |
| TNFRSF9 | TNF receptor superfamily member 9 | ENSG00000049249 |
| ICOS | inducible T cell costimulator | ENSG00000163600 |
| CD80 | CD80 molecule | ENSG00000121594 |
| TIGIT | T cell immunoreceptor with Ig and ITIM domains | ENSG00000181847 |
| CD70 | CD70 molecule | ENSG00000125726 |
| TNFSF9 | TNF superfamily member 9 | ENSG00000125657 |
| CD86 | CD86 molecule | ENSG00000114013 |
| PDCD1 | prephenate dehydratase 1 | ENSG00000188389 |
| LAIR1 | leukocyte associated immunoglobulin like receptor 1 | ENSG00000167613 |
| TNFRSF8 | TNF receptor superfamily member 8 | ENSG00000120949 |
| TNFSF15 | TNF superfamily member 15 | ENSG00000181634 |
| TNFRSF14 | TNF receptor superfamily member 14 | ENSG00000157873 |
| IDO2 | indoleamine 2,3-dioxygenase 2 | ENSG00000188676 |
| CD276 | CD276 molecule | ENSG00000103855 |
| TNFRSF4 | TNF receptor superfamily member 4 | ENSG00000186827 |
| HHLA2 | HERV-H LTR-associating 2 | ENSG00000114455 |
| CD274 | CD274 molecule | ENSG00000120217 |
| HAVCR2 | hepatitis A virus cellular receptor 2 | ENSG00000135077 |
| CD27 | CD27 molecule | ENSG00000139193 |
| LGALS9 | galectin 9 | ENSG00000168961 |
| CD28 | CD28 molecule | ENSG00000178562 |
| TNFRSF25 | TNF receptor superfamily member 25 | ENSG00000215788 |
| VTCN1 | V-set domain containing T cell activation inhibitor 1 | ENSG00000134258 |
| CD44 | CD44 Molecule | ENSG00000026508 |
| TNFSF18 | TNF Superfamily Member 18 | ENSG00000120337 |
| TNFRSF18 | TNF Receptor Superfamily Member 18 | ENSG00000186891 |
| BTNL2 | Butyrophilin Like 2 | ENSG00000204290 |
| CD200R1 | CD200 Receptor 1 | ENSG00000163606 |
| TNFSF4 | TNF Superfamily Member 4 | ENSG00000117586 |
| CD200 | CD200 Molecule | ENSG00000091972 |
| NRP1 | Neuropilin 1 | ENSG00000099250 |
