# Supplementary material for: Identification and Validation of a Prognostic Prediction Model of m6A Regulator-Related LncRNAs in Hepatocellular Carcinoma
Source: Front Mol Biosci. 2021 Dec 20;8:784553. doi: 10.3389/fmolb.2021.784553 (PMC8721125; doi:10.3389/fmolb.2021.784553)
Supplement: Supplementary file 2 [file DataSheet1.docx]

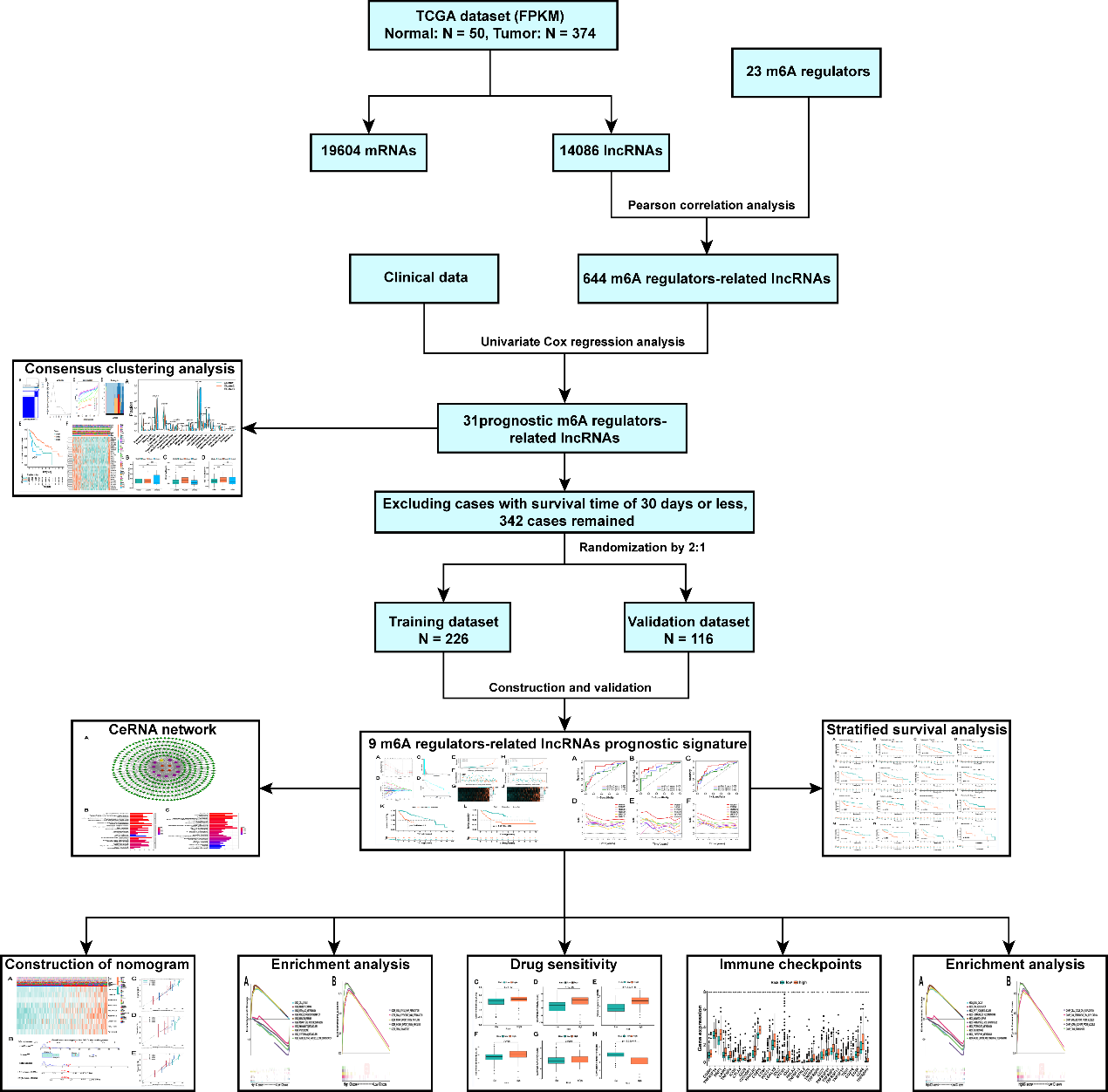


**SUPPLEMENTARY FIGURE 1 |** The flow chart of this study.


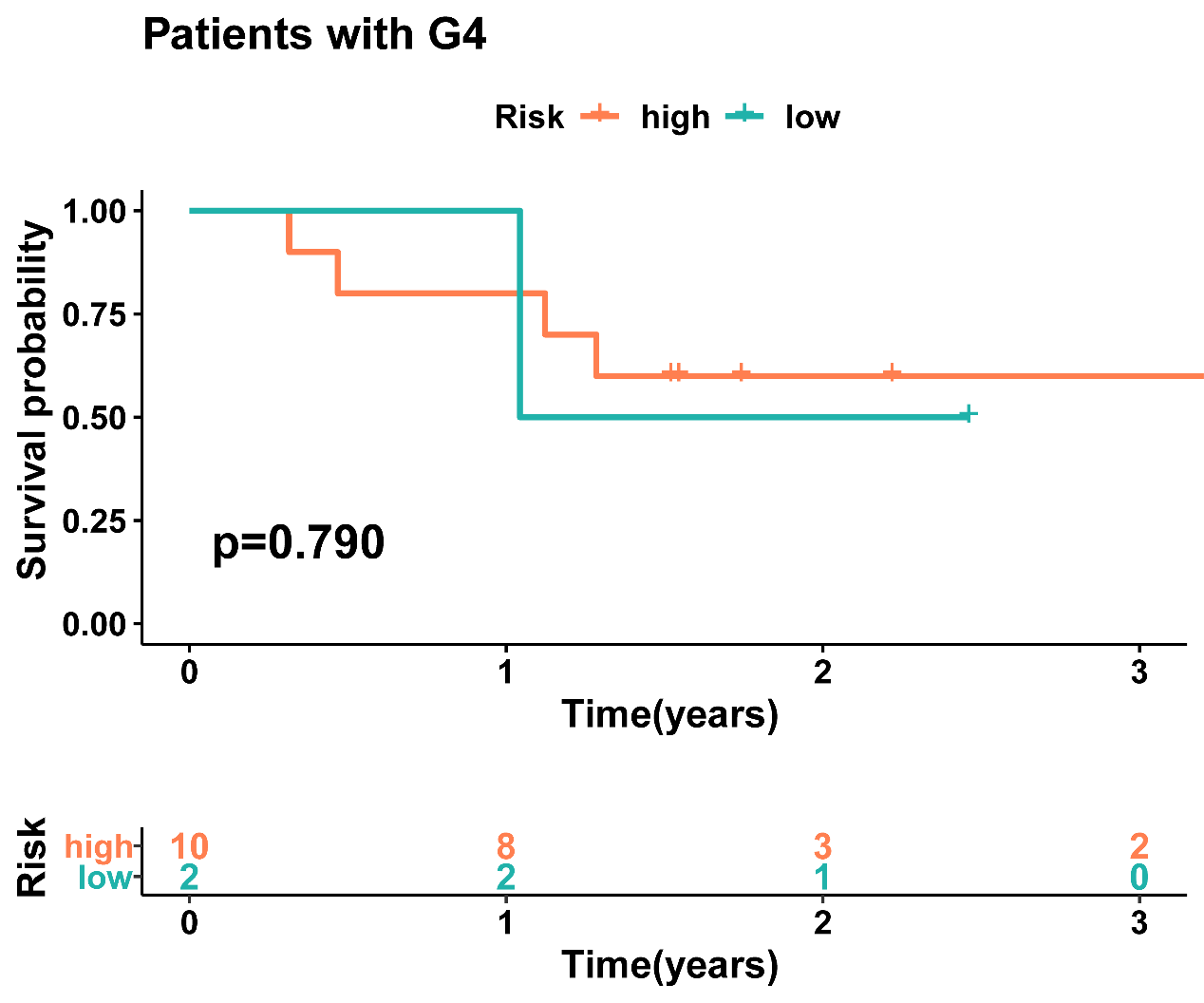


**SUPPLEMENTARY FIGURE 2 |** The KM curve of G4 stage patients with OS according to m6A-9LPS.
